# The plasma proteome reveals distinct signaling pathways associated with PR3-ANCA positive and MPO-ANCA positive vasculitis

**DOI:** 10.3389/fimmu.2025.1600754

**Published:** 2025-06-18

**Authors:** Erik Hellbacher, Vincent van Hoef, Alina Johansson, Ann Knight, Iva Gunnarsson, Annette Bruchfeld, Per Eriksson, Sophie Ohlsson, Solbritt Rantapää-Dahlqvist, Johanna Dahlqvist

**Affiliations:** ^1^ Department of Medical Sciences, Uppsala University, Uppsala, Sweden; ^2^ Rheumatology, Uppsala University Hospital, Uppsala, Sweden; ^3^ National Bioinformatics Support (NBIS), Science for Life Laboratory, Uppsala University, Uppsala, Sweden; ^4^ Department of Medicine, Division of Rheumatology, Karolinska Institutet, Stockholm, Sweden; ^5^ Unit of Rheumatology, Karolinska University Hospital, Stockholm, Sweden; ^6^ Department of Nephrology and Department of Health, Medicine and Caring Sciences, Linköping University, Linköping, Sweden; ^7^ Department of Renal Medicine, Karolinska University Hospital and CLINTEC Karolinska Institutet, Stockholm, Sweden; ^8^ Department of Biomedical and Clinical Sciences, Division of Inflammation and Infection, Linköping University, Linköping, Sweden; ^9^ Department of Clinical Sciences, Division of Nephrology, Lund University and Skåne University Hospital, Lund, Sweden; ^10^ Department of Public Health and Clinical Medicine, Umeå University, Umeå, Sweden

**Keywords:** ANCA, vasculitis, proteinase 3, myeloperoxidase, proteome

## Abstract

**Objective:**

Despite recent advances, the pathophysiological mechanisms underlying anti-neutrophil cytoplasmic antibody (ANCA)-associated vasculitides (AAV) remain incompletely understood, and comparative proteomic analyses of AAV subtypes are lacking. This study aimed to identify key molecular signaling pathways activated in AAV and to elucidate molecular distinctions between AAV with proteinase 3 ANCA (PR3-AAV) and AAV with myeloperoxidase ANCA (MPO-AAV).

**Methods:**

Plasma samples from 41 cases with active PR3-AAV, 24 with active MPO-AAV and 138 population controls were analyzed for 185 proteins using proximity extension assay and Luminex. Differential expression was assessed between PR3-AAV, MPO-AAV and controls using univariate and partial least squares discriminant analyses. Protein-protein interactions and pathway enrichment were explored using STRING and Cytoscape databases.

**Results:**

Compared with controls, 31 proteins were significantly upregulated in PR3-AAV and 29 in MPO-AAV; 18 were shared, whereas 13 and 11 were specific to PR3-AAV and MPO-AAV, respectively. Shared proteins were enriched in general immune pathways, including IL-6 signaling. AAV subgroup-specific proteins were combined with proteins differentiating between PR3-AAV and MPO-AAV in a direct comparison. MMP-1, MMP-9, HGF, and OSM were uniquely upregulated in PR3-AAV, while TNF, TNF-R1/R2, TNFRSF14, and TNFRSF9 were prominent in MPO-AAV. Functional enrichment analyses underscored STAT3 signaling in PR3-AAV and TNF signaling in MPO-AAV.

**Conclusions:**

This study identifies distinct and shared signaling pathways in PR3-AAV and MPO-AAV, highlighting STAT3 and TNF pathways as potential subtype-specific mechanisms. These findings offer insight into AAV pathogenesis and may guide the development of more targeted, less toxic treatments tailored to AAV subtypes.

## Introduction

Granulomatosis with polyangiitis (GPA) and microscopic polyangiitis (MPA) are systemic vasculitides that, along with eosinophilic granulomatosis with polyangiitis (EGPA), constitute the group of anti-neutrophil cytoplasmic autoantibody (ANCA)-associated vasculitides (AAV). GPA and MPA have traditionally been considered as a single entity in clinical studies, due to their shared clinical and pathological characteristics. Both are characterized by necrotizing inflammation in small and medium-sized blood vessels, with frequent involvement of kidneys and lungs. GPA and MPA do, however, also exhibit distinct features; clinically, GPA is associated with more extensive extra-renal organ involvement and higher relapse rates (1, 2), while MPA is characterized by a higher incidence of renal involvement and lung fibrosis (3, 4). Mechanistically, granulomatous inflammation is found in GPA exclusively, a feature associated with bone-destructive capacity (1). The precise mechanisms driving granulomatous inflammation in GPA remain obscure.

Central to the pathophysiology for both AAV subtypes is the loss of tolerance to enzymes within neutrophil granules, leading to the formation of ANCAs. In GPA, ANCA predominantly targets proteinase 3 (PR3), whereas the main target in MPA is myeloperoxidase (MPO), although an overlap between clinical diagnosis and ANCA specificity exists (5). Recent studies suggest that classification of AAV patients based on ANCA serotypes (hereafter called “PR3-AAV” and “MPO-AAV”) provides better differentiation of patient phenotypes, outcomes, and treatment responses compared to classification based on the clinical diagnoses, GPA and MPA (6). AAV classification according to serotypes is further supported by genetic association analyses, where it has been demonstrated that the genetic disease loci in AAV show a stronger association with ANCA serotypes than with the clinical diagnoses GPA and MPA.

The pathophysiological mechanisms driving AAV are complex and remain largely unknown. One hypothesis is that the interactions between ANCAs and neutrophils play a pivotal role in the disease development of AAV. Both PR3-ANCA and MPO-ANCA activate primed neutrophils *in vitro*, triggering the release of reactive oxygen species, lytic enzymes, and the formation of neutrophil extracellular traps (NETs) (7, 8). Together, these processes may contribute to vascular endothelial cell injury and subsequent damage to organ tissue (9).

In addition to neutrophils, several other immune components have been implicated in AAV pathogenesis. Autoreactive B cells are responsible for ANCA production and may contribute to the loss of tolerance and chronic autoimmunity. Dysregulated T cell responses, particularly involving Th1 and Th17 polarization, are thought to promote inflammation and tissue injury through cytokine production, recruitment of inflammatory cells, and support B cell activation (10). Macrophages and dendritic cells may further amplify local inflammation and mediate activation of the complement system, particularly the alternative pathway. This, in turn, contributes to disease progression, with complement component C5a recruiting and priming neutrophils, which may then be activated by, e.g., ANCAs (11–13). Genetic analyses of AAV have pinpointed strong associations with the HLA region for AAV, but, interestingly, PR3-AAV and MPO-AAV are associated with distinct loci within the region. Additionally, PR3-AAV but not MPO-AAV is significantly associated with the genes encoding PR3 and its strongest inhibitor alpha 1-antitrypsin, emphasizing the role of neutrophils and proteolytic enzymes in disease pathogenesis of PR3-AAV (14–16).

Taken together, although recent studies have begun to elucidate the pathophysiological mechanisms underlying AAV, the key cell types and immune signaling pathways driving the inflammatory cascade remain unclear. Moreover, the molecular differences between PR3-AAV and MPO-AAV that contribute to their distinct clinical manifestations are still not fully understood.

Current standard treatments for AAV, including high-dose glucocorticoids with either cyclophosphamide or B cell depletion using rituximab (17), are typically effective in inducing remission in most patients. This broadly immunosuppressive approach is, however, associated with significant risks of severe adverse effects, particularly infections, with potentially fatal consequences (18). It remains unclear whether optimal treatment strategies could differ between PR3-AAV and MPO-AAV. In order to reduce co-morbidities and mortality in AAV, novel targeted therapies with higher precision are needed for both induction and maintenance treatment of PR3-AAV and MPO-AAV.

A better understanding of the pathological processes underlying PR3-AAV and MPO-AAV may guide the development of more targeted, subgroup-specific therapies as well as biomarkers for disease activity. To address this knowledge gap, we hypothesized that a thorough examination of the plasma proteome in patients with PR3-AAV and MPO-AAV during active disease phases would uncover key proteins and molecular pathways activated specifically in these subtypes, offering valuable insights into AAV pathophysiology.

## Materials and methods

### Cases and controls

Plasma samples were obtained from patients diagnosed with PR3-AAV or MPO-AAV across five rheumatological and/or nephrological centers in Sweden (Umeå University Hospital, Lund University Hospital, Karolinska University Hospital, Linköping University Hospital, Uppsala University Hospital). In total, samples were collected from 42 patients with GPA and 25 with MPA fulfilling the classification criteria of the European Medicines Agency algorithm from 2007 for GPA or MPA (19). At the time of sampling, the patients suffered from active disease characterized by systemic manifestations, and had either no ongoing immunosuppressive treatment or a maximum of four days of corticosteroid treatment. For comparison, plasma samples were extracted from Biobank Norr (Biobank Research Unit at Umeå University, Sweden), a population-based sample collection including samples from individuals older than 40 years of age in Västerbotten Sweden, that have volunteered to contribute to the biobank. Samples from 138 individuals matched for age and sex with the AAV patients (2–3 controls/patient) were selected through randomization of individuals included in the biobank. Furthermore, to enable a comparison of the inflammatory protein pattern in PR3-AAV and MPO-AAV to that of other disorders of systemic inflammation, samples from disease controls with active systemic lupus erythematosus (SLE; n=14; Karolinska University Hospital) and rheumatoid arthritis (RA; n=31; Umeå University Hospital), respectively, were analyzed. SLE and RA patient samples were specifically selected for comparison as these disorders are both classified as rheumatic diseases, yet clearly distinct in relation to AAV; SLE is characterized by involvement of a range of organs, similar to AAV, but unlike AAV, formation of immune complexes is a key feature of SLE inflammation. In contrast, RA is mainly characterized by joint inflammation and less involvement of inner organs or blood vessels. Disease controls had no ongoing immunosuppressive treatment, including corticosteroids, at time of sampling.

Clinical data were collected from medical records of all cases and disease controls, including diagnosis, ANCA subtype, sex, age, disease activity scores/indices and levels of serum creatinine, C-reactive protein (CRP) and erythrocyte sedimentation rate (ESR) at the time of sampling. Disease activity in AAV cases was assessed using the Birmingham Vasculitis Activity Score (BVAS), an index validated for different subgroups of AAV and that quantifies systemic vasculitis activity across nine organ systems. The weight of each organ item for the total BVAS is based on the perceived clinical important of each organ system, with manifestations of kidney involvement resulting in high BVAS (20).

Two cases were positive for both PR3-ANCA (high titer) and MPO-ANCA (low titer); they had been diagnosed with GPA and were considered as PR3-ANCA positive in the statistical analyses. To estimate glomerular filtration rate (GFR) of the patients, the chronic kidney disease epidemiology collaboration equation (CKD-EPI) (21) was used, based on creatinine levels. As creatinine levels were not available for the population controls, GFR was estimated for all controls based on population reference values as calculated by van den Brand J. et al. (22). For AAV patients, data on glucocorticoid treatment were collected from medical records. All data and samples were collected after informed and written consent from all individuals. The study complies with the Declaration of Helsinki. The regionally appointed ethics committees approved the research protocol.

### Protein measurements

All plasma samples were analyzed for concentrations of 181 unique proteins using proximity extension assay (PEA) with two Olink pre-designed protein panels named “Inflammation” and “Cardiovascular III” (23), at the SciLifeLab Affinity Proteomics Infrastructure Unit in Uppsala, Sweden. These panels were selected to provide broad coverage of immune-related and vascular-related proteins deemed to be relevant to AAV pathogenesis, capturing proteins involved in inflammation, immune regulation, endothelial activation, and vascular damage. Samples were analyzed in 96-well-plates, with samples from cases and controls randomized across plates. Each plate included internal controls to evaluate technical quality and to normalize values. Assay read-out was provided as Normalized Protein eXpression (NPX), an arbitrary unit on log2-scale where a high value corresponds to a higher protein expression. Samples were analyzed for deviation from the median (>0.3 NPX) of the internal controls as an assessment of quality. Each PEA measurement had a lower detection limit (LOD) calculated based on negative controls; measurements below LOD were removed from further analysis. The distribution of median values of all proteins in all samples were inspected in graphs displaying one boxplot of all proteins per sample, showing comparable median values, not revealing any outliers (**Supplementary Figure 1**).

Additionally, plasma concentrations of proteins TIMP-1, C5a, CCL18 (PARC) and CA15-3 (1), previously identified as potential biomarkers for active AAV disease (24–26) or pulmonary disease (27) were analyzed using Luminex (SciLifeLab Affinity Proteomics Infrastructure Unit, Stockholm, Sweden) as these proteins were not included in the Olink panels. Levels of these proteins were transformed into NPX values and data were analyzed jointly with the Olink proteins.

### Statistical analysis

NPX values were compared between the population control group and PR3-AAV and MPO-AAV, respectively, as well as between PR3-AAV and MPO-AAV. PR3-AAV and MPO-AAV were also compared with RA and SLE patient samples, respectively. For univariate differential expression analysis, an ANOVA F-test was performed to assess variation in protein expression between groups. This was followed by a *post-hoc* test to determine which comparison(s) drove the differential expression, with age and estimated GFR as covariates, using OlinkAnalyze R package (v1.2.4) and the Olink ANOVA posthoc function. *P* values were adjusted for multiple testing using Tukey’s procedure.

Next, NPX values were subject to multivariate partial least squares discriminant analysis (PLS-DA) using the R package mixOmics (v6.14.1). Internal validation was performed using 10 repeated 5-fold cross validations. In the analyses of PR3-AAV and MPO-AAV versus population controls, PLS-DA component 1 alone accounted for 28% and 31% of the variation, respectively, with component 2 explaining 5% for both AAV subgroups (**Supplementary Figures 2A, B**). Hence, the strongest effects of proteins driving separation between groups were found on component 1. In the analysis of PR3-AAV versus MPO-AAV, both component 1 and 2 contributed substantially to the separation, explaining 22% and 11% of the variation, respectively (**Supplementary Figure 2C**). Therefore, in this analysis, both component 1 and 2 were considered in terms of lead proteins driving the separation between groups.

The univariate and multivariate analyses were considered complementary; while ANOVA identifies proteins individually differing between groups, PLS-DA captures combinations of proteins contributing to group separation, and the identification of a protein by both methods was considered supportive of differential expression between groups and indicative of biological relevance.

To account for any potential bias, the effect of ongoing glucocorticoid treatment on protein levels was analyzed using univariate differential expression analysis as described above, comparing treated and non-treated groups with PR3-AAV and MPO-AAV, respectively (significance threshold *P*_adj_ < 0.05, log2 fold change (FC) ≥1).

### Definition of differentially expressed proteins

In the univariate analysis of differentially expressed proteins between AAV subgroups and population and RA/SLE disease controls, as well as between RA/SLE disease controls and population controls, significantly upregulated proteins were defined by a threshold of *P*_adj_ < 0.05 and log2 FC ≥ 1 while downregulated proteins were defined by *P*_adj_ < 0.05 and log2 FC ≤ -1. However, given the relatively small number of cases, the shared inflammatory context for PR3-AAV and MPO-AAV, and our focus on proteins related to inflammation, substantial differences in protein expression were not expected between PR3-AAV and MPO-AAV. Consequently, in this exploratory study, a more lenient significance threshold of *P*_adj_ < 0.10 (with no log2 FC limit) was applied to reduce the risk of overlooking relevant differences due to limited statistical power. While this approach increases the risk of false positive proteins, we seeked to gain further support of the results by evaluating the proteins in the context of complementary analyses, including PLS-DA, comparisons with healthy controls, and protein-protein interaction networks. These analyses helped identify consistent patterns and support the biological relevance of the findings.

### Protein–protein interaction analysis

To explore protein-protein interaction networks, the STRING database (version 11.5) (28) was used. For Homo sapiens, the multiple proteins option was selected, and the analysis was performed using default settings. These combine evidence from experimental data, curated databases, text mining (co-mentioning in publications), co-expression (similar gene expression patterns), and genomic context (neighborhood, gene fusion, and co-occurrence), thereby enabling identification of both direct physical interactions and functional associations. STRING provides a combined confidence score reflecting the overall strength of the integrated evidence, and only interactions with a high confidence score (>0.7) were included in the present study to ensure robust interaction networks. Results were exported to Cytoscape software (version 3.9.1) (29) for network analysis and visualization. To identify hub proteins, defined as proteins with high interconnectivity within protein-protein interaction networks, we employed CytoHubba (version 0.1) (30), a plugin for Cytoscape. The Maximal Clique Centrality (MCC) algorithm was used to evaluate node connectivity within the protein-protein interaction networks (30). Proteins ranking in the top 5 based on their MCC values were classified as hub proteins.

### Protein functional enrichment and signaling pathway analyses

To explore the functional aspects of the proteins with differential expression in PR3-AAV and MPO-AAV compared to population controls and compared to each other, functional enrichment analyses were conducted using the Cytoscape plugin ClueGO (version 2.5.9) (31). These analyses utilized annotations from the Gene Ontology (GO) database (32) to explore biological functions and molecular mechanisms, and data from the Kyoto Encyclopedia of Genes and Genomes (KEGG) (33), Reactome (34), and Wikipathways (35) databases to explore signaling pathways. Only curator-reviewed annotations were used, and the GO term fusion option was applied to reduce redundancy. We set a threshold in the enrichment analysis, requiring at least 3 proteins from the studied sets to be included in a term, accounting for at least 5% of the total proteins associated with that term or pathway for it to be displayed. This threshold aimed to reduce noise from terms with minimal protein representation, ensuring more reliable findings. The “whole genome” (corresponding to the proteins encoded by the genes) was set as the statistical background. Only annotations and pathways with Bonferroni step-down corrected *P* values of less than 0.05 were displayed. Given the use of the whole genome as background and our selection of proteins pre-enriched for inflammation and cardiovascular disease, the reliability of *P* values as a measure of statistical significance in the enrichment analysis is limited. Hence, *P* values were primarily used to rank enrichment terms within and between disease groups.

## Results

### Clinical characteristics of study population

One AAV patient sample was excluded due to poor protein data quality, leaving 66 AAV patient samples for analysis; demographic and clinical characteristics of study participants are summarized in **Table 1** and BVAS in **Supplementary Table 1**. There was no significant difference between patients with PR3-AAV and MPO-AAV in terms of age at diagnosis (mean ± standard deviation (SD), 56 ± 14 and 62 ± 15 years, respectively), proportion of male sex (59% and 60%, respectively), CRP levels (mean ± SD, 49 ± 50 and 26 ± 35 mg/l, respectively), ESR levels (mean ± SD, 54 ± 32 and 57 ± 26 mm, respectively) or disease activity as estimated by BVAS (median (range), 13 (2-29) and 15 (8-27), respectively). Stratification of BVAS by organ showed that PR3-AAV patients more often had involvement of ear-nose-throat (ENT), mucous membranes/eyes, and lungs, whereas renal involvement was more frequent in MPO-AAV, in accordance with what is previously known about AAV (36) (**Supplementary Table 1**). MPO-AAV patients had a significantly lower estimated GFR (mean ± SD, 33 ± 30 ml/min/1.73m²) compared to PR3-AAV patients (75 ± 33 ml/min/1.73m²; *P* < 0.001 (two-sided t test)) and the combined AAV cohort had a significantly lower GFR compared with population controls (**Table 1**; *P* < 0.001). Accordingly, univariate protein analyses were adjusted for estimated GFR.

**Table 1:** Clinical characteristics of patients and controls included in the study after quality control of protein data.

	Cases	Disease controls		Population controls
	AAV	SLE	RA	
Total, n	66	14	31	138
Females, n (%)	26 (41)	12 (86)	23 (74)	56 (41)
Age at sampling, mean (SD)	58 (15)	39 (20)	63 (13)	56 (13)
GPA, n (%)	41 (62)	–	–	–
MPA, n (%)	25 (38)	–	–	–
PR3-ANCA+, n (%)	41 (62)	–	–	–
MPO-ANCA+, n (%)	25 (38)	–	–	–
CKD-EPI eGFR, mean (SD)	60 (38)	97 (30)	89 (18)	93 (20)^a^
CRP, mean (SD)	41 (46)	19 (34)	56 (29)	–
ESR, mean (SD)	55 (30)	62 (23)	78 (13)	–
BVAS/SLEDAI/DAS-28,^b^ mean (SD)	14 (6.3)	15 (7.3)	6.5 (0.76)	–
GC treatment prior to sampling, n (%)	35 (56)	–	–	–

ANCA, anti-neutrophil cytoplasmic autoantibody; AAV, ANCA-associated vasculitis; SLE, Systemic lupus erythematosus; RA, rheumatoid arthritis; SD, standard deviation; GPA, granulomatosis with polyangiitis; MPA, microscopic polyangiitis; PR3, proteinase 3; MPO, myeloperoxidase; BVAS, Birmingham Vasculitis Activity Score; SLEDAI, SLE disease activity index; DAS-28, Disease activity score 28. CKD-EPI, chronic kidney disease epidemiology collaboration; eGFR, estimated glomerular filtration rate; CRP, C-reactive protein; ESR, erythocyte sedimentation rate; ENT, ear-nose-throat; GC, glucocorticoid.

a Based on reference eGFR values for Caucasian population (van den Brand et al., 2011).

b BVAS -AAV; SLEDAI -SLE; DAS-28 -RA.

Approximately 56% of both PR3-AAV and MPO-AAV patients had received glucocorticoid treatment (for no more than four days) prior to sampling. One protein was significantly upregulated in glucocorticoid-treated compared with non-treated PR3-AAV patients (MMP-3: Log2 FC 1.3, *P*_adj_ = 0.0050), and two proteins were downregulated (CCL19: Log2 FC -1.4, *P*_adj_ = 0.022; IL-12B: Log2 FC -1.3, *P*_adj_ = 0.032); there were no significant differences in protein levels between glucocorticoid-treated and non-treated patients with MPO-AAV (data not shown).

### Protein differential expression analysis, AAV vs. population controls

**Figure 1:**
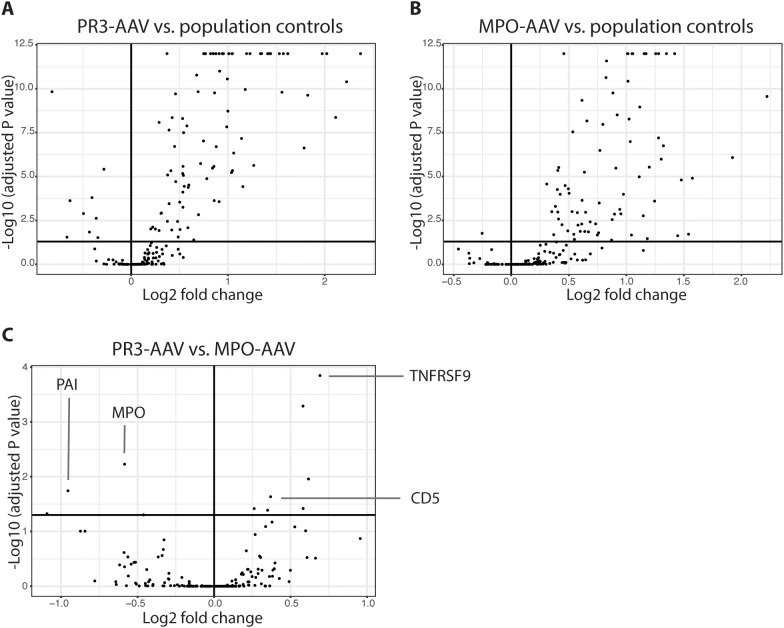
Univariate differential protein analysis. Volcano plots depicting differentially expressed proteins between patients with active PR3-AAV and population controls **(A)**, patients with active MPO-AAV and population controls **(B)** and between patients with PR3-AAV and MPO-AAV **(C)**. Dots represent proteins; upper right square of each plot includes proteins with adjusted P value < 0.05 upregulated in PR3-AAV **(A)**, MPO-AAV **(B)** and MPO-AAV **(C)**, respectively, with upper left squares including proteins with lower levels in these groups. Key proteins differentiating PR3-AAV and MPO-AAV are indicated **(C)**. PR3, proteinase 3; MPO, myeloperoxidase; AAV, antineutrophil cytoplasmic antibody-associated vasculitis.

In total, 185 plasma proteins (**Supplementary Table 2**) were analyzed for differential expression between population controls and patients with active PR3-AAV and MPO-AAV, respectively. In the univariate analysis, 31 significantly upregulated proteins were identified in PR3-AAV and 29 in MPO-AAV; out of these, 18 were common to both subtypes, 13 were specific to PR3-AAV, and 11 were specific to MPO-AAV (**Figures 1A, B**; **Table 2**; **Supplementary Table 3**). For both PR3-AAV and MPO-AAV, IL-6, SIRT2, AXIN1, EN-RAGE and CXCL11 were among the top ten proteins with the largest fold changes. Of the proteins specific to PR3-AAV, CASP-3, MMP-9, TNFSF14, SELP, and GP6 exhibited the largest fold changes, while CXCL9, IGFBP2, TNF-R1, TNF, and CSTB showed the largest fold changes in MPO-AAV. No significantly downregulated proteins were identified in either AAV subtype compared to population controls.

**Table 2:** Differentially expressed proteins in PR3-AAV and MPO-AAV versus population controls.

PR3-AAV				MPO-AAV			
Protein name	Log2 FC (conf.low-conf.high)		*P*_adj_	Protein name	Log2 FC (conf.low-conf.high)		*P*_adj_
IL6	2.4	(1.6-3.2)	<1 x 10¯¹²	NT-proBNP	2.2	(1.2-3.2)	2.7 x 10¯¹^0^
SIRT2	2.2	(1.3-3.2)	3.0 x 10¯¹¹	IL6	1.9	(0.85-3.0)	8.3 x 10¯^7^
AXIN1	2.1	(1.1-3.1)	4.3 x 10¯^9^	EN-RAGE	1.6	(0.61-2.5)	1.2 x 10¯^5^
OSM	2.0	(1.4-2.7)	<1 x 10¯¹²	AXIN1	1.5	(0.13-3.0)	0.019
EN-RAGE	2.0	(1.3-2.7)	<1 x 10¯¹²	CXCL11	1.5	(0.56-2.4)	1.6 x 10¯^5^
STAMBP	1.8	(1.0-2.7)	2.3 x 10¯¹^0^	SIRT2	1.4	(0.10-2.8)	0.024
**CASP-3**	1.8	(0.82-2.8)	2.4 x 10¯^7^	OPN	1.4	(0.91-1.9)	<1 x 10¯¹²
PRTN3	1.6	(1.1-2.1)	<1 x 10¯¹²	CCL18 (PARC)	1.3	(0.83-1.9)	<1 x 10¯¹²
CXCL11	1.6	(0.95-2.3)	<1 x 10¯¹²	ST2	1.3	(0.62-2.0)	1.8 x 10¯^7^
JAM-A	1.6	(0.94-2.2)	<1 x 10¯¹²	**CXCL9**	1.3	(0.57-2.0)	1.0 x 10¯^6^
4E-BP1	1.6	(0.86-2.3)	1.6 x 10¯¹^0^	IL2-RA	1.3	(0.85-1.7)	<1 x 10¯¹²
**MMP-9**	1.5	(0.91-2.2)	<1 x 10¯¹²	MCP-3	1.3	(0.62-1.9)	6.3 x 10¯^8^
ST2	1.4	(0.93-2.0)	<1 x 10¯¹²	**IGFBP-2**	1.3	(0.72-1.8)	<1 x 10¯¹²
**TNFSF14**	1.4	(0.91-1.9)	<1 x 10¯¹²	JAM-A	1.2	(0.38-2.1)	0.00025
CCL18 (PARC)	1.4	(1.0-1.9)	<1 x 10¯¹²	PRTN3	1.2	(0.50-1.9)	2.9 x 10¯^6^
**SELP**	1.3	(0.85-1.8)	<1 x 10¯¹²	STAMBP	1.2	(0.04-2.3)	0.034
MCP-3	1.3	(0.85-1.8)	<1 x 10¯¹²	**TNF-R1**	1.2	(0.77-1.6)	<1 x 10¯¹²
NT-proBNP	1.3	(0.53-2.0)	2.3 x 10¯^6^	CCL23	1.2	(0.77-1.5)	<1 x 10¯¹²
**GP6**	1.2	(0.72-1.7)	<1 x 10¯¹²	OSM	1.1	(0.26-2.0)	0.0017
OPN	1.2	(0.82-1.6)	<1 x 10¯¹²	**TNF**	1.1	(0.59-1.6)	1.1 x 10¯^9^
**CHI3L1**	1.2	(0.65-1.7)	1.1 x 10¯¹^0^	**CSTB**	1.1	(0.43-1.8)	1.0 x 10¯^5^
**MMP-1**	1.2	(0.41-1.9)	3.7 x 10¯^5^	**TNF-R2**	1.1	(0.66-1.4)	<1 x 10¯¹²
**PAI**	1.1	(0.55-1.7)	6.8 x 10¯^8^	**TNFRSF14**	1.1	(0.63-1.5)	<1 x 10¯¹²
**PDGF subunit A**	1.1	(0.48-1.6)	4.5 x 10¯^7^	4E-BP1	1.0	(0.08-2.0)	0.022
**CCL20**	1.0	(0.43-1.7)	4.6 x 10¯^6^	**RETN**	1.0	(0.49-1.6)	1.02 x 10¯^7^
**PGLYRP1**	1.0	(0.66-1.4)	<1 x 10¯¹²	**CCL3**	1.0	(0.53-1.5)	5.3 x 10¯^9^
CCL23	1.0	(0.76-1.3)	<1 x 10¯¹²	**CD40**	1.0	(0.57-1.5)	3.7 x 10¯¹¹
**AZU1**	1.0	(0.42-1.7)	5.9 x 10¯^6^	TIMP-1	1.0	(0.68-1.3)	<1 x 10¯¹²
TIMP-1	1.0	(0.77-1.3)	<1 x 10¯¹²	**U-PAR**	1.0	(0.59-1.4)	<1 x 10¯¹²
IL2-RA	1.0	(0.70-1.3)	<1 x 10¯¹²				
**IL10**	1.0	(0.53-1.5)	1.9 x 10¯^9^				

Proteins highlighted with bold text represent unique differentially expressed proteins for each respective AAV subtype compared to population controls.

AAV subtype compared to population controls.

AAV, ANCA-associated vasculitis; PR3-AAV, proteinase 3 ANCA-positive AAV; MPO-AAV, myeloperoxidase.

ANCA-positive AAV; Log2 FC, log2 fold change; conf.low-conf.high, confidence interval (lower - higher).

*P*_adj_, adjusted *P* value.

**Figure 2:**
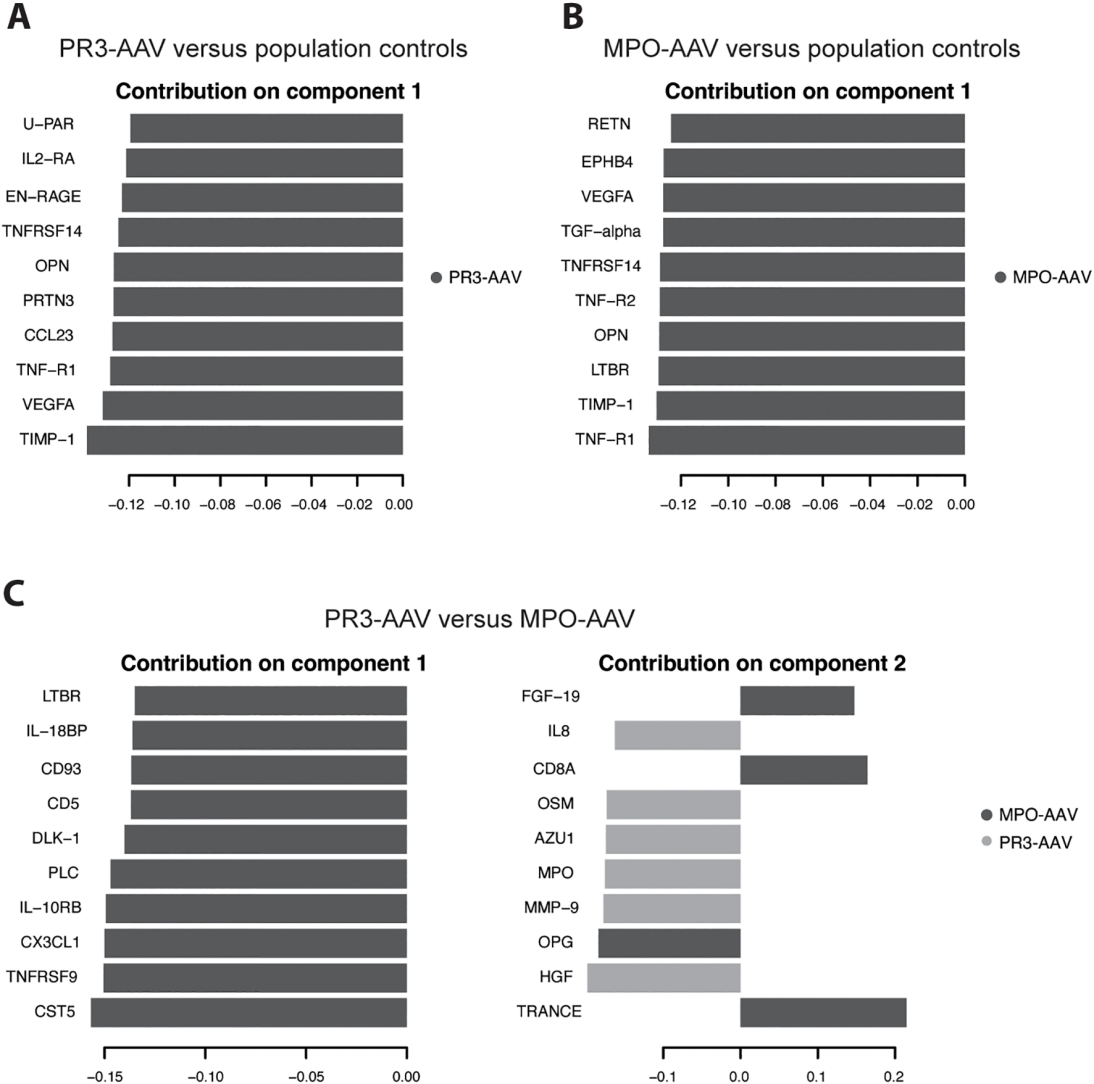
Partial least squares discriminant analysis of 185 proteins. Population controls were analyzed against PR3-AAV cases **(A)** and MPO-AAV cases **(B)**, respectively. **(C)** PR3-AAV cases were analyzed against MPO-AAV cases. Graphs depict lead 10 proteins contributing to the separation of the two groups on component 1 **(A, B)** and component 1 & 2 **(C)**, respectively, with loading weights on X axes. Dark gray/light gray indicate to what group the specified protein is associated. PR3, proteinase 3; MPO, myeloperoxidase; AAV, antineutrophil cytoplasmic antibody-associated vasculitis.

In the multivariate PLS-DA of population controls versus PR3-AAV and MPO-AAV, PR3-AAV and MPO-AAV shared five out of the top ten differentiating proteins on component 1 for each AAV subtype (TIMP-1, VEGFA, TNF-R1, OPN, TNFRSF14), while five proteins were specific for each AAV subtype (PR3-AAV: CCL23, PRTN3, EN-RAGE, IL2-RA, U-PAR; MPO-AAV: LTBR, EPHB4, RETN, TNF-R2, TGF-α; **Figures 2A, B**; **Supplementary Table 4**). In summary, a substantial number of differentially expressed proteins were identified in active inflammatory AAV compared to population controls, including both proteins shared across AAV subtypes and proteins specific to PR3-AAV and MPO-AAV.

### Protein differential expression analysis, PR3-AAV vs. MPO-AAV

In a direct univariate comparison of protein expression levels between PR3-AAV and MPO-AAV, six proteins were significantly upregulated in PR3-AAV, while seven were significantly upregulated in MPO-AAV (**Figure 1C**, **Table 3**; **Supplementary Table 5**). Four additional proteins (SCF, DLK-1, CST5, Notch 3) showed higher levels in MPO-AAV compared to PR3-AAV, owing to a decreased expression in PR3-AAV compared to population controls, rather than an increased expression in MPO-AAV. As a result, these proteins were not considered significantly upregulated in MPO-AAV relative to PR3-AAV.

**Table 3:** Significantly differentially expressed proteins between PR3-AAV and MPO-AAV with comparison to top 10 proteins on PLS-DA components 1 and 2.

Upregulated in PR3-AAV vs MPO-AAV				Upregulated in MPO-AAV vs PR3-AAV			
Protein	Log2 FC (conf.low-conf.high)	*P_adj_*	PLS-DA top 10 protein*	Protein	Log2 FC (conf.low-conf.high)	*P_adj_*	PLS-DA top 10 protein*
MMP-1	1.09 (0.0061-2.2)	0.047	No	TNFRSF9	0.69 (0.22-1.16)	0.00014	Yes
PAI	0.96 (0.086-1.8)	0.018	No	CD8A	0.60 (-0.047-1.2)	0.097	Yes
OSM	0.87 (0.070-1.8)	0.099	Yes	TNF	0.53 (-0.030-1.084)	0.082	No
MMP-9	0.84 (0.068-1.8)	0.099	Yes	CX3CL1	0.38 (-0.012-0.77)	0.067	Yes
MPO	0.59 (0.096-1.07)	0.0059	Yes	CD5	0.37 (0.026-0.71)	0.023	Yes
HGF	0.46 (0.00020-0.92)	0.046	Yes	IL-18BP	0.35 (0.0070-0.69)	0.041	No
				IL-10RB	0.26 (0.0070-0.52)	0.038	Yes

*Among the top 10 discriminating proteins on component 1 or 2 of the PLS-DA comparison between PR3-AAV and MPO-AAV.

AAV, ANCA-associated vasculitis; PR3-AAV, Proteinase 3 ANCA-positive AAV; MPO-AAV, Myeloperoxidase ANCA- positive AAV; PLS-DA, partial least squares discriminant analysis; Log2 FC, log2 fold change; conf.low-conf.high, confidence interval (lower- higher); *P_adj_*., adjusted *P* value.

PLS-DA comparing PR3-AAV and MPO-AAV revealed that all top ten differentiating proteins on component 1 were associated with MPO-AAV, while six of the top ten proteins on component 2 were associated with PR3-AAV and four with MPO-AAV (**Figure 2C**; **Supplementary Table 4**). Ten out of the thirteen proteins significantly upregulated in either PR3-AAV (OSM, MMP-9, MPO, HGF) or MPO-AAV (TNFRSF9, CD8A, CX3CL1, CD5, IL-10RB, IL-18BP), in the univariate analysis were also among the top ten proteins on PLS-DA component 1 or 2. Hence, the PLS-DA confirmed that a majority of the lead proteins identified as differentially expressed between PR3-AAV and MPO-AAV were also fundamental proteins for separating the two AAV subtypes.

### Definition of shared and subtype-specific protein sets for interaction and enrichment analyses

For the subsequent protein-protein interaction and functional enrichment analyses, separate sets of proteins were outlined by combining the results from the univariate and multivariate protein analyses. Firstly, all proteins that were significantly upregulated in both PR3-AAV and MPO-AAV compared to population controls (n = 18), along with any additional proteins from the top ten component 1 proteins of the PLS-DA that differentiated both AAV subtypes from population controls, were jointly defined as “shared AAV proteins” (n total = 21; **Supplementary Table 6**).

Secondly, sets of “PR3-AAV proteins” and “MPO-AAV proteins” were defined as all significantly differentially expressed proteins identified in the univariate comparison between each AAV subtype and population controls (n = 31 and n = 29, respectively) along with any additional proteins from the top ten component 1 proteins of the PLS-DA that differentiated PR3-AAV and MPO-AAV from population controls, (n total PR3-AAV = 35, MPO-AAV = 33; **Supplementary Table 6**).

Thirdly, to further constrain AAV subtype analyses, differentially expressed proteins unique for each AAV subgroup in the comparison with population controls (n PR3-AAV = 13; MPO-AAV = 11) were combined with additional proteins upregulated in PR3-AAV or MPO-AAV, respectively, in the direct comparison between the two AAV subtypes (n PR3-AAV = 3; MPO-AAV = 6). Also, any additional proteins among the top ten component 1 and 2 proteins of the PLS-DA that separated PR3-AAV and MPO-AAV were included (n PR3-AAV = 1; MPO-AAV = 6). These protein sets were defined as “PR3-AAV-specific proteins” and “MPO-AAV-specific proteins”, comprising 17 and 23 proteins, respectively (**Supplementary Table 6**).

### Protein-protein interaction analyses and identification of hub proteins

**Figure 3:**
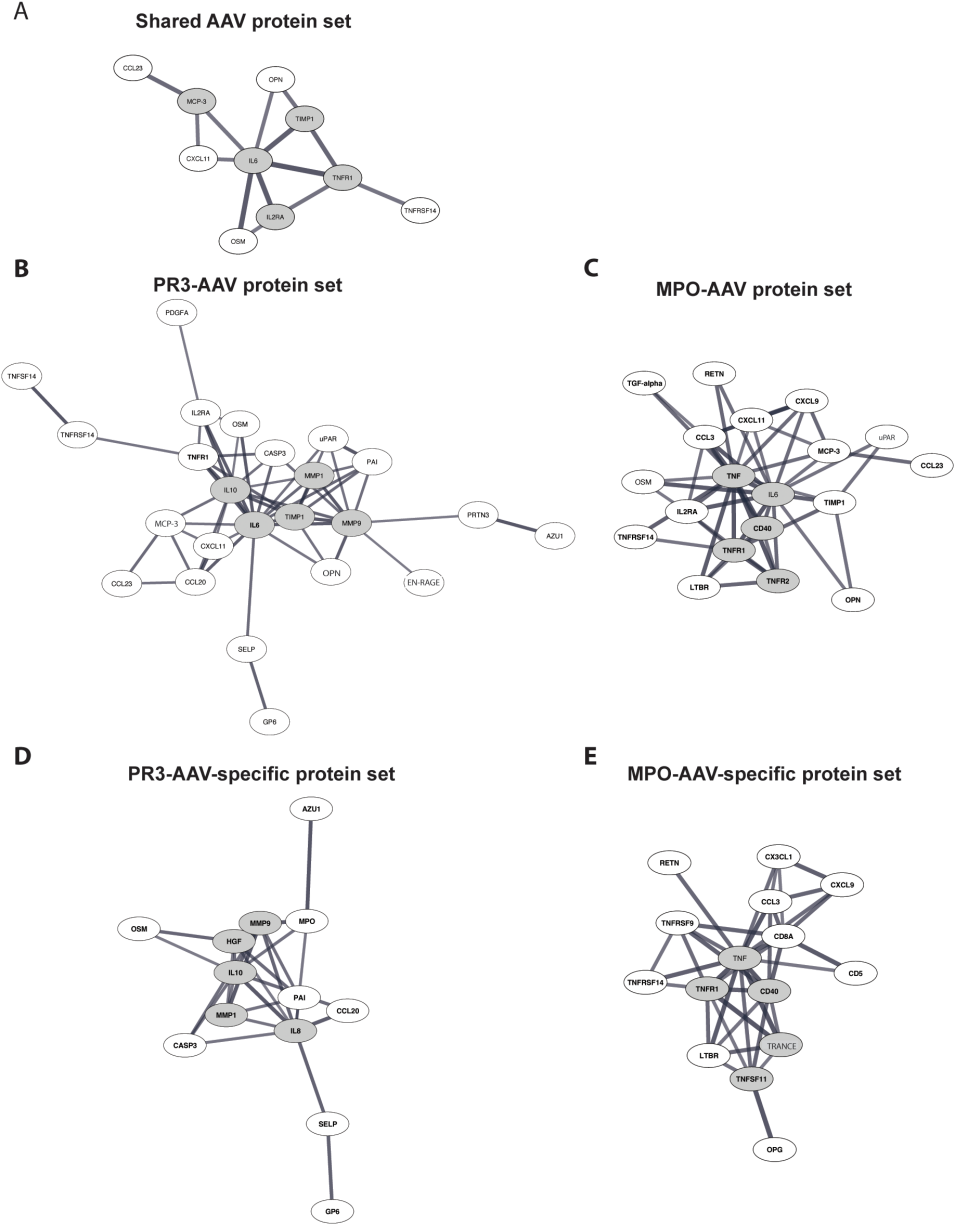
Protein-protein interaction analysis. **(A)** Analysis of proteins upregulated in both PR3-AAV and MPO-AAV. **(B, C)** Analysis of proteins upregulated in PR3-AAV and MPO-AAV, respectively. **(D, E)** Analysis of proteins only upregulated in PR3-AAV and MPO-AAV, respectively. Images depict proteins within the defined protein sets with evidence for interactions according to the STRING database, using a high-confidence interaction score. Proteins with high interconnectivity (“hub proteins”) are indicated by gray ellipses; gray lines represent protein-protein interactions, where line thickness indicates the confidence strength of each interaction. Protein nomenclature from STRING was changed for: MCP-3 (CCL7); OPN (SPP1); EN-RAGE (S100A12); U-PAR (PLAUR); TRANCE (TNFSF11). PR3, proteinase 3; MPO, myeloperoxidase; AAV, antineutrophil cytoplasmic antibody associated vasculitis.

Separate high-confidence protein-protein interaction analyses were conducted for each of the five protein sets using the STRING database, identifying protein interconnectivity and hub proteins (proteins characterized by interaction with multiple protein partners). The vast majority of identified interactions were supported by evidence from at least two of the integrated STRING sources, such as experimental data, curated databases, co-expression, text mining, or genomic context. VEGFA, which was included in the shared AAV, PR3-AAV, and MPO-AAV protein sets, was not available in the STRING database and was therefore excluded. Protein-protein interaction analysis of the shared AAV protein set revealed interconnectivity among ten of the twenty-one proteins (**Figure 3A**). IL-6 emerged as the primary hub protein, followed by TNF-R1, TIMP-1, IL2-RA, and MCP-3 (**Supplementary Table 7**).

In the PR3-AAV protein set, 24 of the 34 proteins were interconnected, with IL-6 identified as the central hub protein, followed by MMP-9, TIMP-1, IL-10, and MMP-1. In the MPO-AAV protein set, interconnectivity was found among 20 of the 32 proteins, with TNF as the main hub protein, followed by IL-6, TNF-R1, TNF-R2, and CD40 (**Figures 3B, C**; **Supplementary Table 7**).

The PR3-AAV-specific protein set displayed interconnectivity among 13 of the 17 included proteins, with CXCL8 identified as the key hub protein, followed by MMP-9, HGF, MMP-1, and IL-10 (**Figure 3D**). Similarly, the 23 MPO-AAV-specific proteins showed interconnectivity among 15 proteins, with TNF as the main hub protein, followed by TNF-R1, CD40, TRANCE, and TNF-R2 (**Figure 3E**; **Supplementary Table 7**).

Overall, the observed interconnectivities reflected both direct and indirect functional associations between proteins, involving shared signaling pathways or related biological processes, as identified through STRING analyses restricted to high-confidence interactions (confidence score >0.7). Several hub proteins recurred between the broader and the more specific AAV subtype protein sets, highlighting their potential central roles in coordinating inflammatory and immune regulatory processes in AAV.

### Functional analysis of shared and subtype-specific AAV protein sets

To explore the biological context of the proteins upregulated in active AAV, the five AAV protein sets were analyzed for functional enrichment in biological processes and signaling pathways. Across the three protein sets “shared AAV proteins”, “PR3-AAV proteins” and “MPO-AAV proteins”, the top functional enrichment terms included broad categories related to inflammation and immune signaling, encompassing cytokine and chemokine activity, immune cell migration and chemotaxis (**Table 4**; **Supplementary Tables 8**-**10**). IL-10 signaling emerged among the top terms for both PR3-AAV and MPO-AAV, whereas IL-4/IL-13 signaling and IL-17 signaling were prominent only for the PR3-AAV protein set. Terms related to the JAK/STAT pathway were present in all three protein sets, but the “Expression of STAT3-upregulated extracellular proteins” pathway involved more proteins and was more significantly enriched in the PR3-AAV protein set compared to the MPO-AAV set.

**Table 4:** All identified biological processes and signaling pathways associated with the shared AAV protein set and the top 15 of the PR3-AAV and MPO-AAV protein sets.

Shared AAV protein set		PR3-AAV protein set		MPO-AAV protein set	
Enrichment term	*P_adj_*	Enrichment term	*P_adj_*	Enrichment term	*P_adj_*
Viral protein interaction with cytokine and cytokine receptor	5.0 x 10¯¹³	Viral protein interaction with cytokine and cytokine receptor	2.2 x 10¯¹^6^	Cytokine-cytokine receptor interaction	1.1 x 10¯¹^9^
Overview of proinflammatory and profibrotic mediators	3.6 x 10¯¹²	Expression of STAT3-upregulated extracellular proteins	6.3 x 10¯¹^4^	Overview of proinflammatory and profibrotic mediators	2.7 x 10¯¹^5^
Expression of STAT3-upregulated extracellular proteins	3.7 x 10¯^8^	Mononuclear cell migration	1.3 x 10¯¹²	Positive regulation of peptidyl-tyrosine phosphorylation	2.5 x 10¯¹^0^
Positive regulation of tyrosine phosphorylation of STAT protein	4.5 x 10¯^8^	Monocyte chemotaxis	8.2 x 10¯¹^0^	Positive regulation of tyrosine phosphorylation of STAT protein	4.3 x 10¯¹^0^
Chemokine activity	1.2 x 10¯^6^	Lymphocyte migration	9.1 x 10¯¹^0^	Neutrophil migration	1.5 x 10¯^9^
Regulation of establishment of endothelial barrier	3.2 x 10¯^6^	Interleukin-4 and Interleukin-13 signaling	1.1 x 10¯^8^	Interleukin-10 signaling	3.4 x 10¯^9^
Eosinophil chemotaxis	1.1 x 10¯^5^	Lymphocyte chemotaxis	2.7 x 10¯^8^	Chemokine activity	6.1 x 10¯^9^
Photodynamic therapy-induced NF-kB survival signaling	1.3 x 10¯^5^	Neutrophil migration	5.5 x 10¯^8^	Toll-like receptor signaling pathway	9.8 x 10¯^9^
Interleukin-10 signaling	1.6 x 10¯^5^	Photodynamic therapy-induced NF-kB survival signaling	5.6 x 10¯^8^	Lung fibrosis	1.8 x 10¯^8^
Thymic stromal lymphopoietin (TSLP) signaling pathway	1.6 x 10¯^5^	Positive regulation of homotypic cell-cell adhesion	1.7 x 10¯^7^	Lymphocyte migration	3.3 x 10¯^8^
		IL-17 signaling pathway	1.7 x 10¯^7^	Prostaglandin signaling	4.2 x 10¯^8^
		Interleukin-10 signaling	1.9 x 10¯^7^	Allograft rejection	1.4 x 10¯^7^
		Chemokine activity	2.9 x 10¯^7^	Chemokine-mediated signaling pathway	1.5 x 10¯^7^
		Burn wound healing	3.2 x 10¯^7^	COVID-19 adverse outcome pathway	1.6 x 10¯^7^

AAV, ANCA-associated vasculitis; PR3-AAV, Proteinase 3 ANCA-positive AAV; MPO-AAV, Myeloperoxidase ANCA-positive AAV; *P_adj_*, adjusted. *P* value.

None of the top functional enrichment terms overlapped between the PR3-AAV-specific protein set and the MPO-AAV-specific protein set (**Table 5**; **Supplementary Tables 11**, **12**). In the PR3-AAV-specific set, the most significantly enriched pathways were “Expression of STAT3-upregulated extracellular proteins”, IL-17 signaling, IL-4/IL-13 signaling and oncostatin M (OSM) signaling. In contrast, the MPO-AAV-specific set was dominated by pathways related to TNF-α signaling, with the “TNF-R2 non-canonical NF-κB” pathway being one of the top enrichment terms.

**Table 5:** All identified biological processes and signaling pathways associated with the PR3-AAV-specific protein set and the top 15 biological processes and signaling pathways of the MPO-AAV-specific protein set.

PR3-AAV-specific protein set		MPO-AAV-specific protein set	
Enrichment term	*P_adj_*	Enrichment term	*P_adj_*
Expression of STAT3-upregulated extracellular proteins	5.1x10¯¹¹	Viral protein interaction with cytokine and cytokine receptor	1.3x10¯¹^4^
Interleukin-4 and Interleukin-13 signaling	2.5x10¯^9^	TNFR2 non-canonical NF-kB pathway	1.5x10¯¹^4^
IL-17 signaling pathway	1.1x10¯^7^	TNFs bind their physiological receptors	5.7x10¯¹²
Malaria	6.3x10¯^7^	cIAP1.2 ubiquitinates NIK in cIAP1.2:TRAF2::TRAF3:NIK	5.2x10¯¹^0^
Lung fibrosis	1.4x10¯^6^	K63polyUb-cIAP1.2 ubiquitinates TRAF3	5.2x10¯¹^0^
Oncostatin M signaling pathway	1.5x10¯^6^	TRAF2 ubiquitinates cIAP1.2 in cIAP1.2:TRAF1:TRAF2:TRAF3:NIK	5.2x10¯¹^0^
IL10 negatively regulates extracellular inflammatory mediators	5.7x10¯^6^	Interleukin-10 signaling	1.6x10¯^8^
Hepatitis C and hepatocellular carcinoma	9.7x10¯^6^	death receptor activity	2.5x10¯^8^
Photodynamic therapy-induced NF-kB survival signaling	1.3x10¯^5^	LTA trimer binds TNF-R1.1B.14	3.6x10¯^8^
Bladder cancer	1.6x10¯^5^	Protesomal degradation of K48polyUb-TRAF3	5.9x10¯^8^
Interleukin-10 signaling	1.6x10¯^5^	TNF receptor superfamily (TNFSF) members mediating non-canonical NF-kB pathway	5.3x10¯^6^
		Neuroinflammatory response	7.8x10¯^6^
		Prostaglandin signaling	2.1x10¯^5^
		Inflammatory response pathway	2.1x10¯^5^
		Chemokine receptors bind chemokines	4.1x10¯^5^

AAV, ANCA-associated vasculitis; PR3-AAV, proteinase 3 ANCA-positive AAV; MPO-AAV, myeloperoxidase ANCA-positive AAV; *P_adj_*, adjusted P value.

Taken together, functional enrichment analyses of proteins upregulated in active AAV revealed inflammatory pathways common for both PR3-AAV and MPO-AAV, but also pathways unique for each AAV subtype.

### Comparisons of protein upregulation between AAV subtypes and disease controls

In comparison with population controls, 33 significantly upregulated proteins were identified in patients with active RA, and 41 in patients with active SLE (**Supplementary Table 13**). Among the eighteen proteins upregulated in both PR3-AAV and MPO-AAV compared with population controls, eleven overlapped with those in RA, and ten with SLE. Upregulation of CCL23 was unique to AAV. Of the thirteen proteins upregulated specifically in PR3-AAV compared to population controls, ten overlapped with those in RA versus population controls, and two with those in SLE; three proteins (AZU1, MMP-9, PGLYRP1) were exclusive to PR3-AAV (**Table 2**; **Supplementary Table 13**). Among the eleven proteins specific for MPO-AAV relative to population controls, one overlapped with RA, and eight with SLE. Three proteins (CD40, CSTB, and TNFRSF14) were unique to MPO-AAV (**Table 2**; **Supplementary Table 13**).

In a direct comparison of PR3-AAV and RA using univariate analysis, one protein was significantly upregulated in PR3-AAV (OSM), and six were upregulated in RA (**Supplementary Table 14**). In comparison with SLE, ten proteins were upregulated in PR3-AAV (AXIN1, SIRT2, STAMBP, OSM, MMP-1, JAM-A, MMP-9, SELP, PAI, PDGF subunit A) and eleven in SLE (**Supplementary Table 14**). In a direct comparison of MPO-AAV and RA, one protein was significantly upregulated in MPO-AAV (NT-proBNP), ten in RA (**Supplementary Table 15**). In comparison with SLE, no upregulated proteins were identified in MPO-AAV, while eight were upregulated in SLE (**Supplementary Table 15**).

In summary, there was a significant overlap in upregulated inflammatory proteins between AAV and RA/SLE, as well as disease-specific upregulation of proteins.

## Discussion

Despite advances in understanding the pathophysiology of AAV, many aspects remain unclear, including the mechanisms driving the distinct manifestations of PR3-AAV and MPO-AAV. Integrating univariate analysis and multivariate PLS-DA of a comprehensive panel of proteins associated with immune response and the cardio-vascular system, we identified upregulated proteins distinguishing active AAV from population controls, as well as PR3-AAV from MPO-AAV. While the biological processes associated with the upregulated proteins were partially shared between the AAV subtypes, the findings also provide clues to distinct immune signaling pathways dominating the inflammatory cascades in PR3-AAV and MPO-AAV, respectively.

Among the proteins differentiating both PR3-AAV and MPO-AAV from population controls, IL-6, TNF-R1, and TIMP-1 emerged as particularly significant across univariate analysis, PLS-DA, hub protein identification and exploration of associated biological processes. IL-6 showed the highest fold change in PR3-AAV and the second-highest in MPO-AAV. TIMP-1, an inhibitor of matrix metalloproteases, has previously been identified as a potential biomarker for distinguishing active AAV from remission, regardless of ANCA subtype (37, 38). Moreover, CCL23, a chemokine involved in the chemotaxis of T cells, monocytes, and neutrophils, emerged as uniquely differentially expressed in AAV, as it was not upregulated in RA or SLE compared to population controls. CCL23, a chemoattractant for T cells and monocytes, has previously been reported to correlate with disease activity in AAV (39), suggesting its potential use as a biomarker for disease monitoring along with its possible role in AAV pathogenesis. Functional enrichment analysis of the shared AAV protein set identified broad inflammatory processes mainly mediated by IL-6 and/or TIMP-1 and STAT signaling. Additionally, in the present study, IL-10 signaling was a lead pathway for PR3-AAV and MPO-AAV together and separately, while previous literature has been ambiguous as to whether IL-10 signaling is restricted to PR3-AAV alone (10, 40).

A primary objective of this study was to identify proteins that differentiate the two AAV subtypes PR3-AAV and MPO-AAV, aiming to uncover distinct activated pathways of pathophysiological relevance. Among the individual proteins in the PR3-AAV-specific protein set, MMP-1, MMP-9, HGF, and OSM were identified as consistently significant in the comparison with MPO-AAV through univariate analysis, PLS-DA, and hub protein identification. Notably, OSM was also significantly upregulated in PR3-AAV compared to RA and SLE, whereas MMP-9 was not upregulated in either of MPO-AAV, RA or SLE, compared with population controls. Protein-protein interaction analysis revealed interconnectivity among these four proteins, suggesting shared underlying regulatory mechanisms, which was supported by their frequent inclusion in top functional enrichment terms.

OSM is a pleiotropic cytokine in the IL-6 family, mediating its effects through activation of the JAK/STAT signaling pathway, particularly involving STAT3 (41). OSM enhances neutrophil adhesion to endothelial cells and chemotaxis, and induce chemokine production by endothelial cells (42, 43). Additionally, neutrophil release of OSM enhances neutrophil and monocyte recruitment, key processes in early neutrophil-driven inflammation in AAV (44). OSM has previously been associated with various autoimmune, cardiovascular, and pulmonary diseases (45, 46). While literature on the role of OSM in AAV is sparse, our results align with a study by Brink M. et al. demonstrating elevated OSM levels in pre-symptomatic PR3-AAV patients, but not MPO-AAV patients, before disease onset (47).

Matrix metalloproteinases (MMPs) have previously been studied as biomarkers of disease activity in AAV, but direct comparisons of MMP expression between PR3-AAV and MPO-AAV are limited (37). MMPs play crucial roles in matrix degradation, modulation of cytokine functions, leukocyte recruitment and, as components of NETs, in exacerbation of vascular and tissue damage (8, 48, 49). Interestingly, MMPs stimulated by OSM have been implicated in both granuloma formation and bone erosion in other diseases, such as tuberculosis, sarcoidosis, and granuloma annulare (50–52). Additionally, MMPs produced by nasal fibroblasts have been linked to the bone erosive capacity of granulomatous inflammation in the ENT region in PR3-AAV (53).

Functional analysis of the PR3-AAV-specific protein set identified the “Expression of STAT3-upregulated extracellular proteins” pathway as the most significantly enriched. This pathway also ranked as the third top enrichment term in the broader PR3-AAV protein set, indicating its significant role not only in differentiating PR3-AAV from MPO-AAV but also in the broader pathophysiological context of PR3-AAV. STAT3 is activated downstream of several cytokines signaling pathways, with the IL-6 family (e.g. OSM) serving as primary activators (54). STAT3 regulates numerous immune processes, involving granulocytes, NK cells, B cell antibody production and memory T cell development, and its dysregulation has been connected with various autoimmune diseases (55). Notably, a recent transcriptomic analysis comparing GPA and MPA, identified STAT3 as a putative upstream regulator of a GPA-specific signature distinct from that of MPA (56).

Among the top enrichment terms associated with the PR3-AAV protein sets were also the signaling pathways of IL-4/IL-13 and IL-17. IL-4, IL-13, and IL-17 themselves were not upregulated in PR3-AAV, possibly due to rapid and transient changes in interleukin levels, but an increase in the receptor subunit IL-17RA was seen in both AAV subtypes. The IL-4/IL-13 pathways are involved in Th2 inflammatory responses and allergic reactions (57). While current evidence supports a predominant Th1 response in AAV (58), there have been reports suggesting an enhanced importance of Th2 response in AAV, e.g. in the nasal mucosa of patients with active GPA (59). Elevations in Th17 cells and IL-17A have been documented in both PR3-AAV and MPO-AAV in previous studies, where IL-17A has been implicated in both neutrophil priming and in granuloma formation in AAV (60).

Taken together, the finding of upregulation of a key set of proteins (OSM, MMP-1, MMP-9, HGF) in PR3-AAV suggests that the regulatory influence of OSM on MMPs via STAT3 signaling may play an important role in PR3-AAV pathogenesis and, specifically, in the granulomatous inflammation characteristic of PR3-AAV/GPA.

For the MPO-AAV-specific protein set, a striking feature was the prevalence of TNF-related proteins; TNF, TNF-R1, TNF-R2, TNFRSF14, TNFRSF9, TRANCE and CD40. TNF itself was upregulated in the direct comparison between MPO-AAV and PR3-AAV and in the comparison with population controls. TNF, TNF-R1, TNF-R2, and CD40 were identified as hub proteins in MPO-AAV-specific and broader protein sets. Upregulation of TNF-R2 has previously been suggested to distinguish MPO-AAV from PR3-AAV (61). Functional enrichment analyses of the MPO-AAV-specific protein set identified several TNF-related pathways among the top enriched terms. Of those, the “TNFR2 non-canonical NF-κB pathway” was the most highly enriched, but “cIAP1,2 ubiquitinates NIK”, “K63polyUb-cIAP1,2 ubiquitinates TRAF3”, and “TRAF2 ubiquitinates cIAP1,2”, all closely linked to TNF and non-canonical NF-κB signaling, were also among the top ranked processes. These pathways describe the roles of cellular inhibitors of apoptosis 1 and 2 (cIAP1/2), TNF receptor-associated factors 2 and 3 (TRAF2/3), and NF-κB-inducing kinase (NIK) in the activation and progression of non-canonical NF-κB signaling (62). This signaling pathway plays a fundamental role in immune regulation, including the function and survival of B and T cells. Dysregulated activation of this pathway has been implicated in the pathogenesis of, e.g., RA and SLE, where the survival and differentiation of autoantibody-producing B cells have been shown to rely on non-canonical NF-κB signaling (62).

Aside from TNF-related proteins, CD8a, and CD5 were upregulated in MPO-AAV compared to PR3-AAV and ranked among the top proteins separating the AAV subtypes in PLS-DA. Protein-protein interaction analysis revealed close interactions among these proteins and TNFRSF9. CD8a and TNFRSF9 play essential roles for CD8+ T cell activation, with CD8a enhancing antigen recognition and TNFRSF9 supporting activation and effector functions (63). CD5 is mainly expressed by T cells and released upon T cell activation (64). These findings underscore the importance of T cell activation, in particular activation of CD8+ T cells, in MPO-AAV.

Collectively, these findings suggest a significant role for TNF signaling in MPO-AAV, particularly via the non-canonical NF-κB pathway, and imply that activation of CD8+ T cells are central to the inflammatory processes.

As expected, both PR3-AAV and MPO-AAV shared a large part of the upregulated immune-related proteins with RA and SLE. Interestingly, among the differentially expressed proteins identified in comparisons with population controls, PR3-AAV shared more proteins with RA, e.g. the MMPs, than with SLE. In contrast, MPO-AAV shared more proteins with SLE than RA.

There are limitations to this study. The sample size was relatively small, in particular for MPO-AAV. The potential impact of glucocorticoid treatment on plasma protein levels warrants consideration. Approximately 56% of both the PR3-AAV and MPO-AAV patients had received glucocorticoids prior to sampling. Comparisons of treated and untreated groups showed significant treatment-related differences in only a few proteins. Taken together, this makes a significant impact of glucocorticoids on the main findings, e.g. protein differences between PR3-AAV and MPO-AAV, less likely, although some influence on protein expression cannot be excluded and, hence, remains a study limitation. In the functional enrichment analyses, use of preselected proteins which have previously been associated with inflammation and cardio-vascular disease, alongside the entire genome as a background, may introduce bias that skews statistical significance. Hence, the *P* values in the enrichment analysis should be interpreted with caution and were primarily used to rank enrichment terms within and between AAV subtypes. Additional limitations include the cross-sectional design, which limits the ability to infer causality or assess temporal changes in protein expression over the disease course. The use of plasma samples may not fully reflect local, tissue-specific processes contributing to disease pathogenesis. Finally, although the study covered a broad set of immune- and vascular-related proteins, the results from the protein interaction and functional enrichment analyses are restricted to the proteins included, and other relevant proteins and pathways may not have been captured.

Conclusively, in this study, we have demonstrated that upregulated immune-related plasma proteins are partially shared and partially unique for active PR3-AAV and MPO-AAV. Both AAV subtypes were associated with broad immune pathways, dominated by IL-6 and STAT signaling. Additionally, PR3-AAV was specifically associated with MMP-, OSM- and STAT3 signaling pathways, while MPO-AAV was associated with enhanced TNF signaling. The findings highlight potential biomarkers for disease activity monitoring in AAV, such as CCL23 and TIMP-1, and suggest that the STAT3 pathway in PR3-AAV and TNF signaling in MPO-AAV may represent subtype-specific therapeutic targets.

The identification of distinct signaling profiles in PR3-AAV and MPO-AAV provides a basis for future studies aiming to improve prognosis prediction and precision treatment strategies in AAV. In a longer perspective, these studies will contribute to reduced organ damage and fatalities due to inflammation or therapeutic adverse effects and improved quality of life for patients with AAV.

## Data Availability

The original contributions presented in the study are included in the article/supplementary material. A metadata record is available at https://doi.org/10.17044/scilifelab.29291642.v1, https://figshare.scilifelab.se/. Further inquiries can be directed to the corresponding author.
