# Supplementary material for: The plasma proteome reveals distinct signaling pathways associated with PR3-ANCA positive and MPO-ANCA positive vasculitis
Source: Front Immunol. 2025 Jun 18;16:1600754. doi: 10.3389/fimmu.2025.1600754 (PMC12213373; doi:10.3389/fimmu.2025.1600754)
Supplement: Supplementary file 1 [file DataSheet1.pdf]

Supplementary Figure 1

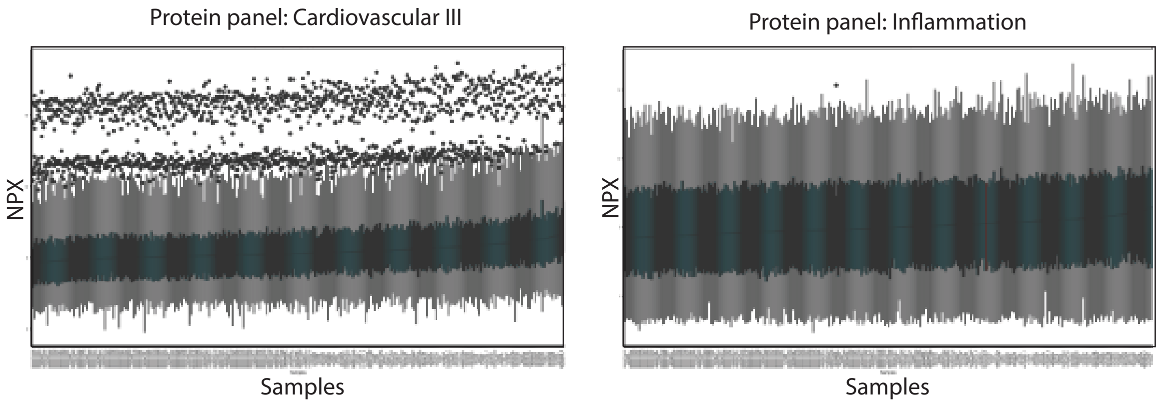

The distribution of median values of all proteins in all samples are depicted with one boxplot/sample along the X axis and NPX (Normalized Protein eXpression) values on the Y axis.

Supplementary Figure 2

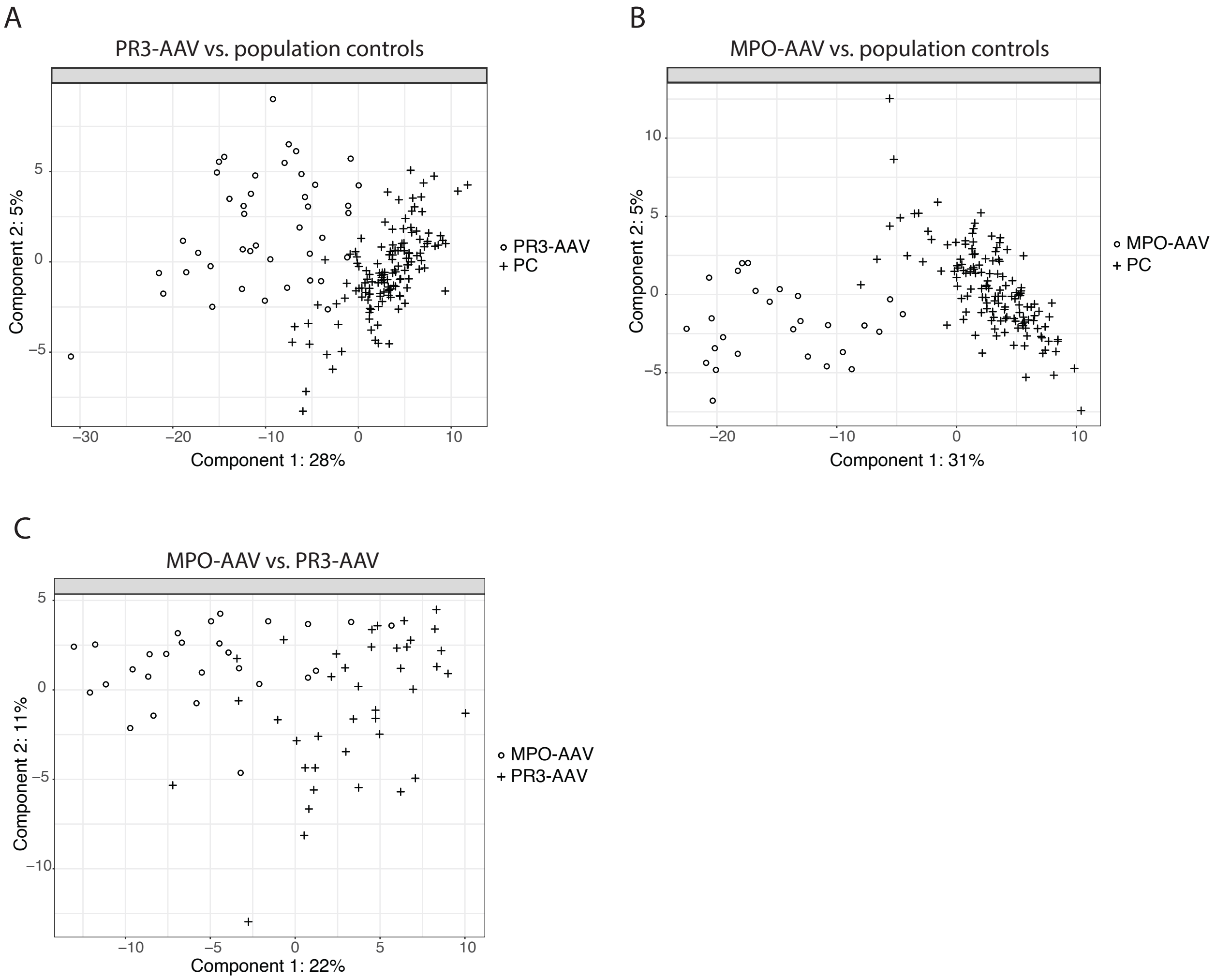

Partial least squares discriminant analysis (PLS-DA) of 185 proteins, separating proteinase 3 positive ANCA-assocated vasculitis (PR3-AAV) cases and poulation controls (PC; **A**), myelo-peroxidase positive AAV (MPO-AAV) cases and poulation controls (**B**), and PR3-AAV and MPO-AAV cases (**C**). Components 1-2 are shown with percentage of variance explained.
