# Supplementary material for: The plasma proteome reveals distinct signaling pathways associated with PR3-ANCA positive and MPO-ANCA positive vasculitis
Source: Front Immunol. 2025 Jun 18;16:1600754. doi: 10.3389/fimmu.2025.1600754 (PMC12213373; doi:10.3389/fimmu.2025.1600754)
Supplement: Supplementary file 2 [file DataSheet2.pdf]

Supplementary tables

"*Plasma proteome profiling identifies distinct signaling pathways associated with PR3-ANCA positive and MPO-ANCA positive vasculitis*"

Hellbacher E. *et al*.

Supplementary table 1. BVAS total score and organ engagement according to BVAS domains.
Supplementary table 2. Details of all 185 proteins analyzed in the study.
Supplementary table 3. Protein differential analysis: PR3-AAV and MPO-AAV versus population controls.
Supplementary table 4. PLS-DA loading scores on component 1 for AAV subtypes vs. population controls and on components 1 and 2 for comparison between the AAV subtypes.
Supplementary table 5. Protein differential analysis: PR3-AAV vs. MPO-AAV.
Supplementary table 6. Sets of differentially expressed proteins based on the univariate and multivariate protein analyses in PR3-AAV and MPO-AAV.
Supplementary table 7. Hub proteins across the AAV protein sets.
Supplementary table 8. Biological processes and signaling pathways associated with the shared AAV protein set.
Supplementary table 9. Biological processes and signaling pathways associated with the PR3-AAV protein set. Top 15 terms according to adjusted P value.
Supplementary table 10. Biological processes and signaling pathways associated with the MPO-AAV protein set. Top 15 terms according to adjusted P value.
Supplementary table 11. Biological processes and signaling pathways associated with the PR3-AAV-specific protein set.
Supplementary table 12. Biological processes and signaling pathways associated with the MPO-AAV-specific protein set.
Supplementary table 13. Proteins with significant differential expression, RA and SLE vs population controls.
Supplementary table 14. Differentially expressed proteins, PR3-AAV vs. RA and SLE.
Supplementary table 15. Differentially expressed proteins, MPO-AAV vs. RA and SLE.

Supplementary table 1. BVAS total score and organ engagement according to BVAS domains.

	**PR3-AAV** (n=41)	**MPO-AAV** (n=25)
BVAS total score, median (range)	13 (2-29)	15 (8-27)
BVAS domains*	(n=38)	(n=22)
General, n (%)	32 (84)	17 (77)
Cutaneous, n (%)	5 (13)	3 (14)
Mucus membrane/eyes, n (%)	9 (24)	0 (0)
ENT, n (%)	24 (63)	2 (9)
Chest, n (%)	21 (55)	4(18)
Abdominal, n (%)	0 (0)	0 (0)
Renal, n (%)	17 (45)	20 (91)
Nervous system, n (%)	3 (8)	3 (14)
Cardiovascular, n (%)	0 (0)	0 (0)

BVAS= Birmingham vasculitis activity score; AAV = ANCA-associated vasculitis; PR3-AAV = Proteinase 3 ANCA-positive AAV; MPO-AAV = Myeloperoxidase ANCA-positive AAV; ENT = ear-nose-throat. *Percentages for the BVAS domains are based on n=38 for PR3-AAV and n=22 for MPO-AAV due to missing data.

Supplementary table 2. Details of all 185 proteins analyzed in the study.

Protein	Full name	OlinkID	UniProt	Olink panel
4E-BP1	Eukaryotic translation initiation factor 4E-binding protein 1	OID00536	Q13541	Olink INFLAMMATION
ADA	Adenosine deaminase	OID00560	P00813	Olink INFLAMMATION
ALCAM	Activated leukocyte cell adhesion molecule	OID00572	Q13740	Olink CARDIOVASCULAR III
AP-N	Aminopeptidase N	OID00611	P15144	Olink CARDIOVASCULAR III
ARTN	Artemin	OID00526	Q5T4W7	Olink INFLAMMATION
AXIN1	Axin-1	OID00487	O15169	Olink INFLAMMATION
AXL	Tyrosine-protein kinase receptor UFO	OID00612	P30530	Olink CARDIOVASCULAR III
AZU1	Azurocidin	OID00597	P20160	Olink CARDIOVASCULAR III
Beta-NGF	Beta-nerve growth factor	OID00519	P01138	Olink INFLAMMATION
BLM hydrolase	Bleomycin hydrolase	OID00581	Q13867	Olink CARDIOVASCULAR III
C5a*	Complement factor 5 alpha	NA	P01031	NA
CA15-3 / MUC-1*	Mucin short variant S1	NA	P15941	NA
CASP-3	Caspase 3	OID00630	P42574	Olink CARDIOVASCULAR III
CASP-8	Caspase 8	OID00550	Q14790	Olink INFLAMMATION
CCL11	C-C motif chemokine 11	OID00505	P51671	Olink INFLAMMATION
CCL15	C-C motif chemokine 15	OID00629	Q16663	Olink CARDIOVASCULAR III
CCL16	C-C motif chemokine 16	OID00654	O15467	Olink CARDIOVASCULAR III
CCL18 / PARC*	C-C motif chemokine 18	NA	P55774	NA
CCL19	C-C motif chemokine 19	OID00513	Q99731	Olink INFLAMMATION
CCL20	C-C motif chemokine 20	OID00556	P78556	Olink INFLAMMATION
CCL23	C-C motif chemokine 23	OID00530	P55773	Olink INFLAMMATION
CCL24	C-C motif chemokine 24	OID00592	O00175	Olink CARDIOVASCULAR III
CCL25	C-C motif chemokine 25	OID00551	O15444	Olink INFLAMMATION
CCL28	C-C motif chemokine 28	OID00539	Q9NRJ3	Olink INFLAMMATION
CCL3	C-C motif chemokine 3	OID00532	P10147	Olink INFLAMMATION
CCL4	C-C motif chemokine 4	OID00498	P13236	Olink INFLAMMATION
CD163	Cluster of differentiation 163	OID00577	Q86VB7	Olink CARDIOVASCULAR III
CD244	Cluster of differentiation 244	OID00477	Q9BZW8	Olink INFLAMMATION
CD40	Cluster of differentiation 40	OID00542	P25942	Olink INFLAMMATION
CD5	Cluster of differentiation 5	OID00531	P06127	Olink INFLAMMATION
CD6	Cluster of differentiation 6	OID00499	P30203	Olink INFLAMMATION
CD8A	Cluster of differentiation 8 alpha	OID05124	P01732	Olink INFLAMMATION
CD93	Cluster of differentiation 93	OID00639	Q9NPY3	Olink CARDIOVASCULAR III
CDCP1	CUB domain-containing protein 1	OID00476	Q9H5V8	Olink INFLAMMATION
CDH5	Cadherin-5	OID00587	P33151	Olink CARDIOVASCULAR III
CHI3L1	Chitinase-3-like protein 1	OID00633	P36222	Olink CARDIOVASCULAR III
CHIT1	Chitotriosidase-1	OID00605	Q13231	Olink CARDIOVASCULAR III
CNTN1	Contactin-1	OID00586	Q12860	Olink CARDIOVASCULAR III
COL1A1	Collagen alpha-1(I) chain	OID00641	P02452	Olink CARDIOVASCULAR III
CPA1	Carboxypeptidase A1	OID00624	P15085	Olink CARDIOVASCULAR III
CPB1	Carboxypeptidase B1	OID00632	P15086	Olink CARDIOVASCULAR III
CSF-1	Macrophage colony-stimulating factor 1	OID00562	P09603	Olink INFLAMMATION
CST5	Cystatin-D	OID00491	P28325	Olink INFLAMMATION
CSTB	Cystatin-B	OID00575	P04080	Olink CARDIOVASCULAR III
CTSD	Cathepsin D	OID00622	P07339	Olink CARDIOVASCULAR III
CTSZ	Cathepsin Z	OID00643	Q9UBR2	Olink CARDIOVASCULAR III
CX3CL1	Chemokine (C-X3-C motif) ligand 1	OID00552	P78423	Olink INFLAMMATION
CXCL1	Chemokine (C-X-C motif) ligand 1	OID00496	P09341	Olink INFLAMMATION
CXCL10	Chemokine (C-X-C motif) ligand 10	OID00535	P02778	Olink INFLAMMATION
CXCL11	Chemokine (C-X-C motif) ligand 11	OID00486	O14625	Olink INFLAMMATION
CXCL16	Chemokine (C-X-C motif) ligand 16	OID00601	Q9H2A7	Olink CARDIOVASCULAR III
CXCL5	Chemokine (C-X-C motif) ligand 5	OID00520	P42830	Olink INFLAMMATION
CXCL6	Chemokine (C-X-C motif) ligand 6	OID00534	P80162	Olink INFLAMMATION
CXCL9	Chemokine (C-X-C motif) ligand 9	OID00490	Q07325	Olink INFLAMMATION
DLK-1	Protein delta homolog 1	OID00598	P80370	Olink CARDIOVASCULAR III
DNER	Delta and Notch-like epidermal growth factor-related receptor	OID01213	Q8NFT8	Olink INFLAMMATION
EGFR	Epidermal growth factor receptor	OID00637	P00533	Olink CARDIOVASCULAR III
EN-RAGE / S100A12	Protein S100-A12	OID00541	P80511	Olink INFLAMMATION
Ep-CAM	Epithelial cell adhesion molecule	OID00610	P16422	Olink CARDIOVASCULAR III
EPHB4	Ephrin type-B receptor 4	OID00569	P54760	Olink CARDIOVASCULAR III
FABP4	Fatty acid-binding protein	OID00589	P15090	Olink CARDIOVASCULAR III
FAS	Tumor necrosis factor receptor superfamily member 6	OID00615	P25445	Olink CARDIOVASCULAR III
FGF-19	Fibroblast growth factor 19	OID00545	O95750	Olink INFLAMMATION
FGF-21	Fibroblast growth factor 21	OID00512	Q9NSA1	Olink INFLAMMATION
FGF-23	Fibroblast growth factor 23	OID00507	Q9GZV9	Olink INFLAMMATION
FGF-5	Fibroblast growth factor 5	OID00509	P12034	Olink INFLAMMATION
Flt3L	Fms-related tyrosine kinase 3 ligand	OID00533	P49771	Olink INFLAMMATION
Gal-3	Galectin-3	OID00578	P17931	Olink CARDIOVASCULAR III
Gal-4	Galectin-4	OID00626	P56470	Olink CARDIOVASCULAR III
GDF-15	Growth/differentiation factor 15	OID00595	Q99988	Olink CARDIOVASCULAR III
GDNF	Glial cell line-derived neurotrophic factor	OID00475	P39905	Olink INFLAMMATION
GP6	Platelet glycoprotein VI	OID05026	Q9HCN6	Olink CARDIOVASCULAR III
GRN	Progranulin	OID00579	P28799	Olink CARDIOVASCULAR III
HGF	Hepatocyte growth factor	OID00522	P14210	Olink INFLAMMATION
ICAM-2	Intercellular adhesion molecule 2	OID00646	P13598	Olink CARDIOVASCULAR III
IFN-gamma	Interferon gamma	OID05547	P01579	Olink INFLAMMATION
IGFBP-1	Insulin-like growth factor-binding protein 1	OID00604	P08833	Olink CARDIOVASCULAR III
IGFBP-2	Insulin-like growth factor-binding protein 2	OID00650	P18065	Olink CARDIOVASCULAR III
IGFBP-7	Insulin-like growth factor-binding protein 7	OID00638	Q16270	Olink CARDIOVASCULAR III
IL-1 alpha	Interleukin-1 alpha	OID00493	P01583	Olink INFLAMMATION
IL10	Interleukin-10	OID00528	P22301	Olink INFLAMMATION
IL-10RA	Interleukin-10 receptor alpha	OID00508	Q13651	Olink INFLAMMATION
IL-10RB	Interleukin-10 receptor beta	OID00515	Q08334	Olink INFLAMMATION
IL-12B	Interleukin-12 beta	OID00523	P29460	Olink INFLAMMATION
IL13	Interleukin-13	OID00525	P35225	Olink INFLAMMATION
IL-15RA	Interleukin-15 receptor A	OID00514	Q13261	Olink INFLAMMATION
IL-17A	Interleukin-17 A	OID00485	Q16552	Olink INFLAMMATION
IL-17C	Interleukin-17 C	OID00483	Q9P0M4	Olink INFLAMMATION
IL-17RA	Interleukin-17 receptor A	OID00566	Q96F46	Olink CARDIOVASCULAR III
IL18	Interleukin-18	OID00501	Q14116	Olink INFLAMMATION
IL-18BP	Interleukin-18 binding protein	OID00640	O95998	Olink CARDIOVASCULAR III
IL-18R1	Interleukin-18 receptor 1	OID00517	Q13478	Olink INFLAMMATION
IL-1RT1	Interleukin-1 receptor type 1	OID00613	P14778	Olink CARDIOVASCULAR III

IL-1RT2	Interleukin-1 receptor type 2	OID00627	P27930	Olink CARDIOVASCULAR III
IL2	Interleukin-2	OID00495	P60568	Olink INFLAMMATION
IL-20	Interleukin-20	OID00537	Q9NYY1	Olink INFLAMMATION
IL-20RA	Interleukin-20 receptor alpha	OID00489	Q9UHF4	Olink INFLAMMATION
IL-22 RA1	Interleukin-21 receptor alpha 1	OID00516	Q8N6P7	Olink INFLAMMATION
IL-24	Interleukin-24	OID00524	Q13007	Olink INFLAMMATION
IL2-RA	Interleukin-2 receptor alpha	OID00570	P01589	Olink CARDIOVASCULAR III
IL-2RB	Interleukin-2 receptor beta	OID00492	P14784	Olink INFLAMMATION
IL33	Interleukin-33	OID00543	O95760	Olink INFLAMMATION
IL4	Interleukin-4	OID00546	P05112	Olink INFLAMMATION
IL5	Interleukin-5	OID00559	P05113	Olink INFLAMMATION
IL6	Interleukin-6	OID00482	P05231	Olink INFLAMMATION
IL-6RA	Interleukin-6 receptor alpha	OID00602	P08887	Olink CARDIOVASCULAR III
IL7	Interleukin-7	OID00478	P13232	Olink INFLAMMATION
IL8	Interleukin-8	OID00471	P10145	Olink INFLAMMATION
ITGB2	Integrin beta-2	OID00565	P05107	Olink CARDIOVASCULAR III
JAM-A	Junctional adhesion molecule A	OID00625	Q9Y624	Olink CARDIOVASCULAR III
KLK6*	Kallikrein-6	OID00647	Q92876	Olink CARDIOVASCULAR III
LAP TGF-beta-1	Transforming growth factor beta-1 proprotein	OID00480	P01137	Olink INFLAMMATION
LDL receptor	Low-density lipoprotein receptor	OID00564	P01130	Olink CARDIOVASCULAR III
LIF	Leukemia inhibitory factor	OID00547	P15018	Olink INFLAMMATION
LIF-R	Leukemia inhibitory factor receptor	OID00511	P42702	Olink INFLAMMATION
LTBR	Tumor necrosis factor receptor superfamily member 3	OID00583	P36941	Olink CARDIOVASCULAR III
MB	Myoglobin	OID00616	P02144	Olink CARDIOVASCULAR III
MCP-1	Monocyte chemoattractant protein 1	OID00484	P13500	Olink INFLAMMATION
MCP-2	Monocyte chemoattractant protein 2	OID00549	P80075	Olink INFLAMMATION
MCP-3	Monocyte chemoattractant protein 3	OID00474	P80098	Olink INFLAMMATION
MCP-4	Monocyte chemoattractant protein 4	OID00504	Q99616	Olink INFLAMMATION
MEPE	Matrix extracellular phosphoglycoprotein	OID00132	Q9NQ76	Olink CARDIOVASCULAR III
MMP-1	Matrix metalloproteinase 1	OID00510	P03956	Olink INFLAMMATION
MMP-10	Matrix metalloproteinase 10	OID00527	P09238	Olink INFLAMMATION
MMP-2	Matrix metalloproteinase 2	OID00614	P08253	Olink CARDIOVASCULAR III
MMP-3	Matrix metalloproteinase 3	OID00644	P08254	Olink CARDIOVASCULAR III
MMP-9	Matrix metalloproteinase 9	OID00568	P14780	Olink CARDIOVASCULAR III
MPO	Myeloperoxidase	OID00600	P05164	Olink CARDIOVASCULAR III
Notch 3	Neurogenic locus notch homolog protein 3	OID00584	Q9UM47	Olink CARDIOVASCULAR III
NRTN	Neurturin	OID00548	Q99748	Olink INFLAMMATION
NT-3	Neurotrophin-3	OID00554	P20783	Olink INFLAMMATION
NT-proBNP	N-terminal prohormone of brain natriuretic peptide	OID00131	NA	Olink CARDIOVASCULAR III
OPG	Tumor necrosis factor receptor superfamily member 11B	OID00479	O00300	Olink INFLAMMATION
OPN	Osteopontin	OID00621	P10451	Olink CARDIOVASCULAR III
OSM	Oncostatin M	OID00494	P13725	Olink INFLAMMATION
PAI	Plasminogen activator inhibitor 1	OID00591	P05121	Olink CARDIOVASCULAR III
PCSK9	Proprotein convertase subtilisin/kexin type 9	OID00619	Q8NBP7	Olink CARDIOVASCULAR III
PDGF subunit A	Platelet-derived growth factor subunit A	OID00648	P04085	Olink CARDIOVASCULAR III
PD-L1	Programmed cell death 1 ligand 1	OID00518	Q9NZQ7	Olink INFLAMMATION
PECAM-1	Platelet endothelial cell adhesion molecule	OID00652	P16284	Olink CARDIOVASCULAR III
PGLYRP1	Peptidoglycan recognition protein 1	OID00623	O75594	Olink CARDIOVASCULAR III
PI3	Elafin	OID00609	P19957	Olink CARDIOVASCULAR III
PLC	Basement membrane-specific heparan sulfate proteoglycan core protein	OID00582	P98160	Olink CARDIOVASCULAR III
PON3	Serum paraoxonase/lactonase 3	OID00642	Q15166	Olink CARDIOVASCULAR III
PRTN3	Proteinase 3	OID00618	P24158	Olink CARDIOVASCULAR III
PSP-D	Pulmonary surfactant-associated protein D	OID00608	P35247	Olink CARDIOVASCULAR III
RARRES2	Retinoic acid receptor responder protein 2	OID00645	Q99969	Olink CARDIOVASCULAR III
RETN	Resistin	OID00603	Q9HD89	Olink CARDIOVASCULAR III
SCF	Kit ligand	OID00500	P21583	Olink INFLAMMATION
SCGB3A2	Secretoglobin family 3A member 2	OID00636	Q96PL1	Olink CARDIOVASCULAR III
SELE	E-selectin	OID00596	P16581	Olink CARDIOVASCULAR III
SELP	P-selectin	OID00574	P16109	Olink CARDIOVASCULAR III
SHPS-1	Tyrosine-protein phosphatase non-receptor type substrate 1	OID00628	P78324	Olink CARDIOVASCULAR III
SIRT2	NAD-dependent protein deacetylase sirtuin-2	OID00538	Q8IXJ6	Olink INFLAMMATION
SLAMF1	Signaling lymphocytic activation molecule	OID00502	Q13291	Olink INFLAMMATION
SPON1	Spondin-1	OID00599	Q9HCB6	Olink CARDIOVASCULAR III
ST1A1	Sulfotransferase 1A1	OID00557	P50225	Olink INFLAMMATION
ST2	Interleukin-1 receptor-like 1	OID00634	Q01638	Olink CARDIOVASCULAR III
STAMBP	STAM-binding protein	OID00558	O95630	Olink INFLAMMATION
TFF3	Trefoil factor 3	OID00573	Q07654	Olink CARDIOVASCULAR III
TFPI	Tissue factor pathway inhibitor	OID00590	P10646	Olink CARDIOVASCULAR III
TGF-alpha	Transforming growth factor alpha	OID00503	P01135	Olink INFLAMMATION
TIMP-1*	Tissue inhibitor of metalloproteinases 1	NA	P01033	NA
TIMP4	Tissue inhibitor of metalloproteinases 4	OID00585	Q99727	Olink CARDIOVASCULAR III
TLT-2	Trem-like transcript 2 protein	OID00588	Q5T2D2	Olink CARDIOVASCULAR III
TNF	Tumor necrosis factor	OID05548	P01375	Olink INFLAMMATION
TNFB	Tumor necrosis factor beta	OID00561	P01374	Olink INFLAMMATION
TNF-R1	Tumor necrosis factor receptor 1	OID00649	P19438	Olink CARDIOVASCULAR III
TNF-R2	Tumor necrosis factor receptor 2	OID00567	P20333	Olink CARDIOVASCULAR III
TNFRSF10C	Tumor necrosis factor receptor superfamily member 10c	OID00594	O14798	Olink CARDIOVASCULAR III
TNFRSF14	Tumor necrosis factor receptor superfamily member 14	OID00563	Q92956	Olink CARDIOVASCULAR III
TNFRSF9	Tumor necrosis factor receptor superfamily member 9	OID00553	Q07011	Olink INFLAMMATION
TNFSF13B	Tumor necrosis factor superfamily member 13 beta	OID00617	Q9Y275	Olink CARDIOVASCULAR III
TNFSF14	Tumor necrosis factor superfamily member 14	OID00506	O43557	Olink INFLAMMATION
t-PA	Tissue-type plasminogen activator	OID00635	P00750	Olink CARDIOVASCULAR III
TR	Transferrin receptor protein 1	OID00593	P02786	Olink CARDIOVASCULAR III
TRAIL	Tumor necrosis factor ligand superfamily member 10	OID00488	P50591	Olink INFLAMMATION
TRANCE	Tumor necrosis factor ligand superfamily member 11	OID00521	O14788	Olink INFLAMMATION
TR-AP	Tartrate-resistant acid phosphatase type 5	OID00606	P13686	Olink CARDIOVASCULAR III
TSLP	Thymic stromal lymphopoietin	OID00497	Q969D9	Olink INFLAMMATION
TWEAK	Tumor necrosis factor ligand superfamily member 12	OID00555	O43508	Olink INFLAMMATION
uPA	Urokinase-type plasminogen activator	OID00481	P00749	Olink INFLAMMATION
U-PAR	Urokinase plasminogen activator surface receptor	OID00620	Q03405	Olink CARDIOVASCULAR III
VEGFA	Vascular endothelial growth factor A	OID00472	P15692	Olink INFLAMMATION
vWF	Von Willebrand Factor	OID00651	P04275	Olink CARDIOVASCULAR III

UniProt = universal protein resource identifier, provides detailed protein sequence and functional information
OlinkID = unique identifier for each protein as listed in the Olink proteomic platform.
NA = not applicable; * = proteins analyzed using Luminex as these proteins were not included in the Olink panels.

Supplementary table 3. Protein differential analysis: PR3-AAV and MPO-AAV versus population controls.

AAV subtype	Protein	Log2 FC	conf.low	conf.high	P_{adj}
**PR3-AAV**					
	IL6	2.37	1.59	3.15	1.0 × 10^-12
	OSM	2.02	1.38	2.67	1.0 × 10^-12
	EN-RAGE	1.97	1.27	2.68	1.0 × 10^-12
	PRTN3	1.62	1.11	2.13	1.0 × 10^-12
	CXCL11	1.62	0.95	2.28	1.0 × 10^-12
	JAM-A	1.57	0.94	2.20	1.0 × 10^-12
	MMP-9	1.53	0.91	2.15	1.0 × 10^-12
	ST2	1.44	0.93	1.95	1.0 × 10^-12
	TNFSF14	1.42	0.91	1.93	1.0 × 10^-12
	CCL18_PARC	1.41	1.03	1.79	1.0 × 10^-12
	SELP	1.35	0.85	1.84	1.0 × 10^-12
	MCP-3	1.34	0.85	1.82	1.0 × 10^-12
	GP6	1.23	0.72	1.73	1.0 × 10^-12
	OPN	1.19	0.82	1.56	1.0 × 10^-12
	PGLYRP1	1.05	0.66	1.43	1.0 × 10^-12
	CCL23	1.04	0.76	1.33	1.0 × 10^-12
	TIMP-1	1.01	0.77	1.25	1.0 × 10^-12
	IL2-RA	1.01	0.70	1.32	1.0 × 10^-12
	HGF	0.95	0.64	1.27	1.0 × 10^-12
	TNF-R1	0.94	0.65	1.24	1.0 × 10^-12
	CCL3	0.94	0.58	1.30	1.0 × 10^-12
	VEGFA	0.91	0.62	1.20	1.0 × 10^-12
	CCL16	0.88	0.52	1.23	1.0 × 10^-12
	CD40	0.87	0.55	1.20	1.0 × 10^-12
	U-PAR	0.85	0.54	1.15	1.0 × 10^-12
	MPO	0.82	0.49	1.15	1.0 × 10^-12
	TNF-R2	0.77	0.48	1.05	1.0 × 10^-12
	TNFRSF14	0.76	0.46	1.07	1.0 × 10^-12
	TGF-alpha	0.75	0.45	1.05	1.0 × 10^-12
	PD-L1	0.75	0.49	1.01	1.0 × 10^-12
	CSF-1	0.37	0.23	0.51	1.0 × 10^-12
	RETN	0.91	0.52	1.31	9.9 × 10^-12
	IL-18R1	0.68	0.38	0.97	1.7 × 10^-11
	PECAM-1	0.99	0.56	1.43	2.8 × 10^-11
	SIRT2	2.23	1.25	3.20	4.0 × 10^-11
	CHI3L1	1.18	0.65	1.71	1.1 × 10^-10
	SCF	-0.82	-1.19	-0.45	1.5 × 10^-10
	TNFRSF10C	0.69	0.38	1.00	1.5 × 10^-10
	4E-BP1	1.56	0.86	2.26	1.6 × 10^-10
	IGFBP-2	0.86	0.47	1.25	1.7 × 10^-10
	CXCL16	0.46	0.25	0.67	1.9 × 10^-10
	STAMBP	1.83	1.00	2.65	2.3 × 10^-10
	IL10	1.00	0.53	1.47	1.9 × 10^-9
	AXIN1	2.11	1.09	3.14	4.3 × 10^-9
	EPHB4	0.42	0.22	0.63	4.4 × 10^-9
	LTBR	0.53	0.27	0.79	5.0 × 10^-9
	SPON1	0.29	0.15	0.43	8.2 × 10^-9
	TLT-2	0.58	0.29	0.86	1.3 × 10^-8
	CSTB	0.99	0.49	1.48	1.5 × 10^-8
	RARRES2	0.39	0.19	0.59	2.2 × 10^-8
	CCL15	0.54	0.27	0.82	3.2 × 10^-8
	PAI	1.14	0.55	1.73	6.8 × 10^-8
	MMP-10	0.75	0.36	1.14	9.4 × 10^-8
	TFPI	0.45	0.21	0.69	1.9 × 10^-7
	IL8	0.88	0.41	1.35	2.0 × 10^-7
	CASP-3	1.79	0.82	2.75	2.4 × 10^-7
	PDGF subunit A	1.06	0.48	1.64	4.6 × 10^-7
	CCL4	0.72	0.31	1.13	1.8 × 10^-6
	NT-proBNP	1.27	0.53	2.00	2.3 × 10^-6
	BLM hydrolase	0.54	0.22	0.85	2.6 × 10^-6
	GDF-15	0.85	0.36	1.35	2.7 × 10^-6
	IL7	0.84	0.35	1.33	3.3 × 10^-6
	DNER	-0.28	-0.45	-0.12	3.8 × 10^-6
	CCL20	1.05	0.43	1.67	4.6 × 10^-6
	GDNF	0.41	0.17	0.65	4.7 × 10^-6
	AZU1	1.04	0.42	1.66	5.9 × 10^-6
	LIF	0.53	0.21	0.85	6.7 × 10^-6
	IL-6RA	0.38	0.15	0.60	8.1 × 10^-6
	CD163	0.54	0.21	0.87	8.6 × 10^-6
	MCP-4	0.78	0.30	1.26	1.3 × 10^-5
	IL-17RA	0.46	0.17	0.75	1.9 × 10^-5
	TR	0.60	0.22	0.98	3.0 × 10^-5
	LAP TGF-beta-1	0.55	0.20	0.90	3.6 × 10^-5
	MMP-1	1.15	0.41	1.90	3.7 × 10^-5
	TNF	0.59	0.21	0.97	4.2 × 10^-5
	TIMP4	0.53	0.18	0.89	7.7 × 10^-5
	CNTN1	-0.41	-0.68	-0.13	0.00016
	vWF	0.86	0.26	1.46	0.00023
	PON3	-0.63	-1.07	-0.19	0.00024
	MMP-3	0.91	0.27	1.55	0.00027
	ADA	0.50	0.15	0.85	0.00029
	CTSD	0.39	0.11	0.67	0.00035
	TR-AP	0.53	0.15	0.92	0.00056
	ICAM-2	0.31	0.08	0.55	0.0012
	IL-18BP	0.31	0.07	0.54	0.0013
	Flt3L	-0.49	-0.87	-0.12	0.0013
	CXCL9	0.70	0.16	1.23	0.0015
	ITGB2	-0.36	-0.65	-0.08	0.0024
	Gal-3	0.37	0.07	0.68	0.0036
	IL18	0.48	0.09	0.87	0.0036

IL-1RT1	0.22	0.03	0.41	0.0076
FABP4	0.59	0.08	1.09	0.0082
PLC	0.20	0.03	0.38	0.0096
SLAMF1	0.25	0.03	0.47	0.0098
t-PA	0.50	0.07	0.94	0.01
IL-24	0.42	0.05	0.79	0.011
IL-1RT2	0.29	0.03	0.54	0.012
DLK-1	-0.43	-0.82	-0.05	0.015
MCP-2	0.49	0.03	0.94	0.028
FGF-19	-0.66	-1.29	-0.04	0.028
TNFB	-0.34	-0.67	-0.02	0.03
CXCL10	0.65	0.01	1.28	0.04
OPG	0.21	0.00	0.42	0.06
AXL	0.20	-0.01	0.41	0.086
MCP-1	0.36	-0.02	0.75	0.088
PCSK9	0.26	-0.02	0.55	0.11
TRANCE	-0.38	-0.80	0.05	0.13
SCGB3A2	0.49	-0.06	1.03	0.14
CDCP1	0.32	-0.04	0.68	0.14
IL-15RA	0.17	-0.02	0.36	0.14
Gal-4	0.26	-0.05	0.58	0.21
IGFBP-7	0.18	-0.04	0.40	0.21
IL-10RB	0.14	-0.03	0.32	0.23
AP-N	0.18	-0.04	0.41	0.23
FAS	0.23	-0.05	0.51	0.24
CXCL1	0.50	-0.12	1.13	0.25
TFF3	0.32	-0.08	0.73	0.26
GRN	0.17	-0.04	0.39	0.26
CXCL6	0.42	-0.13	0.98	0.31
TNFSF13B	0.29	-0.10	0.68	0.36
Beta-NGF	0.14	-0.05	0.32	0.4
ST1A1	0.54	-0.20	1.28	0.4
IL-17C	0.25	-0.10	0.61	0.42
TNFRSF9	0.23	-0.09	0.55	0.43
SHPS-1	0.18	-0.07	0.43	0.44
CD244	0.18	-0.08	0.44	0.5
IL-1 alpha	0.14	-0.07	0.35	0.52
CD6	-0.23	-0.58	0.12	0.53
CST5	-0.19	-0.48	0.10	0.56
EGFR	-0.09	-0.23	0.05	0.59
IL-17A	0.28	-0.17	0.73	0.64
IL-12B	-0.36	-0.95	0.22	0.65
IL-20RA	0.16	-0.10	0.42	0.65
CCL28	0.16	-0.10	0.43	0.67
KLK6	-0.16	-0.42	0.11	0.72
MEPE	0.21	-0.15	0.58	0.72
CD93	0.12	-0.09	0.32	0.74
PI3	0.22	-0.17	0.60	0.78
CD5	0.13	-0.11	0.36	0.8
CTSZ	0.13	-0.12	0.38	0.83
CCL19	0.33	-0.33	1.00	0.87
SELE	0.18	-0.18	0.54	0.88
ARTN	0.13	-0.15	0.40	0.91
TSLP	0.13	-0.15	0.40	0.92
COL1A1	-0.13	-0.41	0.15	0.93
uPA	-0.09	-0.30	0.11	0.93
Notch 3	-0.11	-0.35	0.13	0.93
IGFBP-1	0.31	-0.40	1.02	0.94
Ep-CAM	-0.28	-0.94	0.37	0.95
MB	0.16	-0.23	0.54	0.96
CASP-8	0.22	-0.34	0.79	0.97
CPB1	-0.18	-0.65	0.30	0.98
LIF-R	0.07	-0.12	0.27	0.98
IL-22 RA1	0.11	-0.19	0.41	0.98
CHIT1	0.26	-0.44	0.95	0.98
IL-20	0.10	-0.17	0.37	0.98
FGF-21	0.34	-0.63	1.30	0.99
TWEAK	-0.08	-0.31	0.15	0.99
IL33	0.05	-0.11	0.21	0.99
LDL receptor	-0.12	-0.49	0.25	0.99
IFN-gamma	-0.25	-1.08	0.57	1.0
CD8A	0.14	-0.30	0.58	1.0
TRAIL	-0.08	-0.34	0.19	1.0
IL-10RA	0.10	-0.26	0.46	1.0
IL2	0.03	-0.09	0.15	1.0
CXCL5	0.21	-0.66	1.09	1.0
CX3CL1	0.06	-0.20	0.33	1.0
CDH5	-0.04	-0.24	0.16	1.0
PSP-D	-0.09	-0.59	0.41	1.0
CA15-3_MUC-1	-0.08	-0.53	0.37	1.0
CCL24	0.09	-0.44	0.61	1.0
IL13	-0.06	-0.46	0.34	1.0
NT-3	0.04	-0.28	0.36	1.0
IL-2RB	0.04	-0.35	0.44	1.0
CCL25	-0.03	-0.32	0.26	1.0
FGF-23	0.05	-0.45	0.56	1.0
CPA1	-0.04	-0.57	0.48	1.0
IL4	-0.04	-0.48	0.41	1.0
CCL11	0.02	-0.29	0.33	1.0
FGF-5	0.01	-0.14	0.16	1.0
NRTN	0.01	-0.20	0.22	1.0
ALCAM	0.01	-0.16	0.18	1.0
MMP-2	0.00	-0.26	0.25	1.0
C5a	0.00	-0.43	0.42	1.0
IL5	-0.01	-0.49	0.48	1.0

**MPO-AAV**	OPN	1.42	0.91	1.93	1.0 × 10^-12
	CCL18_PARC	1.35	0.83	1.87	1.0 × 10^-12
	IL2-RA	1.28	0.85	1.71	1.0 × 10^-12
	IGFBP-2	1.25	0.72	1.79	1.0 × 10^-12
	TNF-R1	1.17	0.77	1.58	1.0 × 10^-12
	CCL23	1.15	0.77	1.54	1.0 × 10^-12
	TNF-R2	1.06	0.66	1.45	1.0 × 10^-12
	TNFRSF14	1.05	0.63	1.47	1.0 × 10^-12
	TIMP-1	1.01	0.68	1.35	1.0 × 10^-12
	U-PAR	1.01	0.59	1.43	1.0 × 10^-12
	CSF-1	0.46	0.27	0.65	1.0 × 10^-12
	LTBR	0.83	0.47	1.18	2.6 × 10^-12
	PD-L1	0.82	0.46	1.18	2.3 × 10^-11
	CD40	1.01	0.57	1.46	3.7 × 10^-11
	VEGFA	0.88	0.49	1.28	1.7 × 10^-10
	NT-proBNP	2.22	1.21	3.23	2.7 × 10^-10
	EPHB4	0.61	0.33	0.90	4.6 × 10^-10
	TNF	1.12	0.59	1.64	1.1 × 10^-9
	TNFRSF9	0.92	0.48	1.36	3.1 × 10^-9
	CCL3	1.02	0.53	1.52	5.3 × 10^-9
	IL-18BP	0.66	0.34	0.98	6.8 × 10^-9
	TLT-2	0.80	0.40	1.19	1.1 × 10^-8
	RARRES2	0.54	0.26	0.81	2.9 × 10^-8
	MCP-3	1.28	0.62	1.94	6.3 × 10^-8
	RETN	1.03	0.49	1.58	1.0 × 10^-7
	ST2	1.32	0.62	2.03	1.8 × 10^-7
	TGF-alpha	0.77	0.35	1.19	3.3 × 10^-7
	IL6	1.92	0.85	3.00	8.3 × 10^-7
	CXCL9	1.30	0.57	2.04	1.0 × 10^-6
	PRTN3	1.20	0.50	1.90	2.9 × 10^-6
	PLC	0.41	0.17	0.65	3.0 × 10^-6
	PGLYRP1	0.91	0.38	1.44	3.3 × 10^-6
	IL-10RB	0.40	0.16	0.64	4.5 × 10^-6
	CCL15	0.64	0.26	1.02	5.7 × 10^-6
	CSTB	1.11	0.43	1.79	1.0 × 10^-5
	EN-RAGE	1.57	0.61	2.54	1.2 × 10^-5
	CXCL11	1.48	0.56	2.39	1.6 × 10^-5
	SPON1	0.31	0.11	0.50	2.7 × 10^-5
	SLAMF1	0.47	0.17	0.77	3.2 × 10^-5
	CD5	0.50	0.17	0.82	5.0 × 10^-5
	IL-1RT1	0.40	0.14	0.66	5.5 × 10^-5
	GDNF	0.50	0.17	0.84	9.0 × 10^-5
	CXCL16	0.43	0.14	0.71	0.0001
	IL10	0.98	0.32	1.63	0.0001
	TNFRSF10C	0.62	0.19	1.04	0.00023
	JAM-A	1.25	0.38	2.12	0.00025
	MMP-10	0.76	0.22	1.30	0.00032
	CD93	0.38	0.11	0.66	0.0005
	GP6	0.94	0.25	1.64	0.00074
	IL-17RA	0.53	0.13	0.92	0.001
	IL-15RA	0.35	0.09	0.62	0.001
	IGFBP-7	0.40	0.10	0.71	0.001
	TIMP4	0.65	0.16	1.13	0.0011
	Gal-4	0.57	0.14	1.01	0.0011
	CHI3L1	0.95	0.23	1.68	0.0013
	GDF-15	0.90	0.21	1.58	0.0014
	OSM	1.15	0.26	2.03	0.0017
	ICAM-2	0.41	0.09	0.74	0.0026
	TNFSF14	0.88	0.18	1.58	0.0028
	TFF3	0.69	0.13	1.24	0.0038
	CD8A	0.73	0.13	1.34	0.005
	MEPE	0.61	0.11	1.12	0.005
	CX3CL1	0.44	0.07	0.81	0.0056
	CCL16	0.58	0.10	1.06	0.0058
	HGF	0.49	0.06	0.93	0.012
	PI3	0.60	0.07	1.13	0.013
	CCL4	0.64	0.07	1.21	0.013
	PECAM-1	0.67	0.07	1.27	0.014
	SELP	0.76	0.08	1.44	0.016
	DNER	-0.25	-0.48	-0.02	0.017
	AXIN1	1.54	0.13	2.95	0.019
	CDCP1	0.54	0.05	1.04	0.019
	CTSZ	0.37	0.03	0.71	0.021
	FABP4	0.75	0.06	1.44	0.021
	4E-BP1	1.04	0.08	2.01	0.022
	SIRT2	1.45	0.10	2.79	0.024
	STAMBP	1.18	0.04	2.32	0.034
	IL18	0.55	0.02	1.08	0.038
	CCL20	0.87	0.02	1.73	0.041
	CST5	0.39	0.00	0.79	0.053
	IL-6RA	0.30	-0.01	0.62	0.072
	IL-24	0.48	-0.03	0.98	0.081
	LIF-R	0.25	-0.01	0.52	0.082
	CXCL10	0.79	-0.08	1.66	0.12
	Flt3L	-0.46	-0.97	0.06	0.14
	EGFR	-0.17	-0.36	0.02	0.14
	TR	0.45	-0.07	0.97	0.16
	CASP-3	1.15	-0.18	2.47	0.16
	SHPS-1	0.29	-0.05	0.63	0.18
	IL-18R1	0.34	-0.07	0.74	0.20
	CD163	0.37	-0.08	0.82	0.23
	TNFB	-0.37	-0.82	0.08	0.23
	GRN	0.24	-0.06	0.53	0.25
	MMP-9	0.69	-0.17	1.54	0.25

FGF-23	0.55	-0.14	1.24	0.26
TR-AP	0.42	-0.11	0.96	0.27
MCP-1	0.42	-0.11	0.95	0.28
TNFSF13B	0.39	-0.15	0.93	0.40
TFPI	0.24	-0.09	0.57	0.42
LDL receptor	-0.36	-0.87	0.15	0.45
ITGB2	-0.27	-0.67	0.12	0.47
AXL	0.20	-0.09	0.49	0.47
SCGB3A2	0.52	-0.24	1.28	0.49
Notch 3	0.23	-0.11	0.56	0.50
IGFBP-1	0.65	-0.33	1.63	0.55
MMP-3	0.57	-0.30	1.45	0.56
CHIT1	0.62	-0.34	1.58	0.58
ALCAM	0.15	-0.08	0.38	0.61
CTSD	0.24	-0.14	0.62	0.63
FGF-5	0.13	-0.08	0.33	0.64
Gal-3	0.25	-0.16	0.67	0.68
ADA	0.29	-0.19	0.78	0.68
IL33	0.13	-0.09	0.34	0.73
FAS	0.22	-0.16	0.61	0.74
ARTN	0.22	-0.16	0.59	0.75
IL8	0.37	-0.28	1.01	0.77
BLM hydrolase	0.24	-0.19	0.67	0.77
vWF	0.46	-0.36	1.28	0.77
AZU1	0.48	-0.38	1.33	0.78
PDGF subunit A	0.44	-0.36	1.24	0.79
TSLP	-0.21	-0.58	0.17	0.80
IL-20	0.20	-0.17	0.57	0.80
PON3	-0.32	-0.93	0.28	0.82
MB	0.28	-0.25	0.81	0.83
LAP TGF-beta-1	0.25	-0.23	0.73	0.85
CCL19	0.47	-0.45	1.38	0.86
MPO	0.23	-0.23	0.69	0.86
SELE	0.25	-0.25	0.75	0.89
PSP-D	0.34	-0.35	1.02	0.89
NT-3	0.21	-0.23	0.64	0.90
TWEAK	-0.15	-0.46	0.17	0.91
CASP-8	-0.36	-1.14	0.41	0.91
IL-20RA	0.16	-0.20	0.52	0.95
CNTN1	-0.16	-0.54	0.22	0.96
CD244	0.15	-0.21	0.51	0.96
IL7	0.28	-0.40	0.95	0.97
KLK6	0.15	-0.22	0.51	0.97
CD6	-0.19	-0.68	0.29	0.97
SCF	-0.20	-0.71	0.30	0.97
MCP-4	0.26	-0.41	0.92	0.98
IL-17A	0.24	-0.38	0.85	0.98
Beta-NGF	0.10	-0.16	0.36	0.98
LIF	0.17	-0.27	0.61	0.98
IL4	0.23	-0.38	0.84	0.98
IL-12B	0.30	-0.50	1.11	0.98
PCSK9	0.14	-0.25	0.54	0.99
MCP-2	0.23	-0.41	0.86	0.99
TRAIL	-0.13	-0.49	0.23	0.99
C5a	-0.20	-0.79	0.39	0.99
OPG	0.09	-0.20	0.39	0.99
IL-10RA	0.16	-0.34	0.66	0.99
IL-17C	0.14	-0.35	0.63	1.0
uPA	-0.08	-0.37	0.20	1.0
CDH5	0.08	-0.20	0.36	1.0
DLK-1	0.15	-0.38	0.68	1.0
CCL11	-0.11	-0.54	0.31	1.0
CCL28	0.09	-0.28	0.45	1.0
CCL25	0.09	-0.31	0.50	1.0
PAI	0.19	-0.63	1.00	1.0
CPB1	-0.14	-0.79	0.52	1.0
IL-2RB	-0.11	-0.66	0.44	1.0
FGF-19	-0.17	-1.04	0.69	1.0
IFN-gamma	-0.21	-1.34	0.92	1.0
CXCL5	-0.22	-1.43	0.98	1.0
CA15-3_MUC-1	0.11	-0.51	0.73	1.0
CPA1	-0.13	-0.85	0.59	1.0
IL5	0.12	-0.55	0.79	1.0
NRTN	0.05	-0.24	0.34	1.0
COL1A1	0.06	-0.33	0.45	1.0
FGF-21	-0.15	-1.48	1.18	1.0
IL-1RT2	-0.04	-0.39	0.31	1.0
MMP-2	0.04	-0.32	0.40	1.0
IL-22 RA1	-0.05	-0.46	0.37	1.0
CCL24	-0.08	-0.80	0.65	1.0
t-PA	0.06	-0.54	0.67	1.0
IL-1 alpha	-0.02	-0.31	0.26	1.0
AP-N	0.02	-0.29	0.33	1.0
CXCL1	0.05	-0.81	0.92	1.0
MMP-1	0.06	-0.96	1.08	1.0
Ep-CAM	0.05	-0.85	0.95	1.0
IL2	0.01	-0.16	0.18	1.0
ST1A1	0.03	-0.99	1.05	1.0
TRANCE	0.00	-0.58	0.59	1.0
IL13	0.00	-0.55	0.55	1.0
CXCL6	0.00	-0.76	0.75	1.0

PR3-AAV = Proteinase 3-ANCA positive vasculitis; MPO-AAV = Myeloperoxidase-ANCA positive vasculitis.;
Log2 FC = log2 fold change; P_{adj} = adjusted P value; Conf.low-conf.high = confidence interval (lower- higher).

Supplementary table 4. PLS-DA loading scores on component 1 for AAV subtypes vs. population controls and on components 1 and 2 for comparison between the AAV subtypes.

PR3-AAV vs population controls		MPO-AAV vs population controls		PR3-AAV vs MPO-AAV			
Component 1		Component 1		Component 1		Component 2	
Protein	Loading score	Protein	Loading score	Protein	Loading score	Protein	Loading score
TIMP-1	-0.139	TNF-R1	-0.134	CST5	-0.157	HGF	-0.198
VEGFA	-0.132	TIMP-1	-0.130	TNFRSF9	-0.151	OPG	-0.184
TNF-R1	-0.128	LTBR	-0.129	CX3CL1	-0.150	MMP-9	-0.178
CCL23	-0.127	OPN	-0.129	IL-10RB	-0.149	MPO	-0.176
PRTN3	-0.127	TNF-R2	-0.129	PLC	-0.147	AZU1	-0.174
OPN	-0.127	TNFRSF14	-0.129	DLK-1	-0.140	OSM	-0.173
TNFRSF14	-0.125	TGF-alpha	-0.127	CD5	-0.137	IL8	-0.163
EN-RAGE	-0.123	VEGFA	-0.127	CD93	-0.137	TSLP	-0.147
IL2-RA	-0.121	EPHB4	-0.127	IL-18BP	-0.136	PGLYRP1	-0.146
U-PAR	-0.119	RETN	-0.124	LTBR	-0.135	LIF	-0.136
TGF-alpha	-0.119	U-PAR	-0.124	IGFBP-7	-0.134	TNFSF14	-0.131
CD40	-0.119	PLC	-0.121	EPHB4	-0.134	PRTN3	-0.121
MCP-3	-0.118	IL2-RA	-0.121	TFF3	-0.132	CD163	-0.121
TNF-R2	-0.118	CD40	-0.120	KLK6	-0.131	IL-1RT2	-0.120
ST2	-0.118	CCL23	-0.120	IL-15RA	-0.130	CHI3L1	-0.119
LTBR	-0.117	CSF-1	-0.118	CCL15	-0.128	IL-1 alpha	-0.118
PGLYRP1	-0.117	CCL15	-0.118	TNFRSF14	-0.127	MMP-2	-0.116
HGF	-0.116	TNFRSF9	-0.117	Gal-4	-0.127	IL-18R1	-0.114
TNFSF14	-0.116	NT-proBNP	-0.116	TNF-R2	-0.127	PCSK9	-0.114
PD-L1	-0.115	IL-18BP	-0.116	FGF-5	-0.127	MMP-1	-0.109
CSF-1	-0.115	PD-L1	-0.115	CTSZ	-0.126	GRN	-0.109
IL6	-0.114	CXCL16	-0.114	NT-proBNP	-0.126	CTSD	-0.108
OSM	-0.113	CSTB	-0.114	TNF-R1	-0.125	SPON1	-0.103
MPO	-0.113	CX3CL1	-0.114	MEPE	-0.122	TNFRSF10C	-0.103
CXCL16	-0.112	PGLYRP1	-0.114	IGFBP-2	-0.121	U-PAR	-0.102
CCL16	-0.112	IL-10RB	-0.113	PI3	-0.119	TGF-alpha	-0.097
CCL18 (PARC)	-0.111	CD93	-0.112	Notch 3	-0.117	CCL28	-0.096
EPHB4	-0.109	IL-15RA	-0.112	FGF-23	-0.114	vWF	-0.094
RETN	-0.109	CD5	-0.112	LIF-R	-0.111	CCL16	-0.094
SELP	-0.107	PRTN3	-0.111	FABP4	-0.110	MMP-3	-0.091
CCL3	-0.107	IGFBP-2	-0.111	IL-1RT1	-0.105	FAS	-0.088
CCL15	-0.106	IGFBP-7	-0.110	MB	-0.104	GDF-15	-0.087
CSTB	-0.105	FABP4	-0.110	SCF	-0.103	CASP-8	-0.085
JAM-A	-0.104	ST2	-0.109	OPN	-0.099	AXL	-0.084
GP6	-0.102	MEPE	-0.109	CCL25	-0.099	IL6	-0.084
SPON1	-0.101	SPON1	-0.109	U-PAR	-0.097	CSTB	-0.084
IL-18R1	-0.100	GDF-15	-0.105	SHPS-1	-0.096	Beta-NGF	-0.083
MMP-9	-0.098	CCL3	-0.104	CSF-1	-0.096	CCL18 (PARC)	-0.082
4E-BP1	-0.098	TFF3	-0.103	FAS	-0.096	RETN	-0.082
IL10	-0.097	TIMP4	-0.103	CD40	-0.095	IL-17C	-0.081
TFPI	-0.097	GDNF	-0.103	GDF-15	-0.095	IL-24	-0.078
GDNF	-0.097	EN-RAGE	-0.101	FGF-19	-0.093	TNF-R1	-0.077
TNFRSF10C	-0.097	IL-1RT1	-0.101	TIMP4	-0.093	CCL24	-0.077
CXCL11	-0.096	CHI3L1	-0.101	ALCAM	-0.091	PD-L1	-0.076
STAMBP	-0.096	CST5	-0.101	SPON1	-0.091	TNF-R2	-0.075
CHI3L1	-0.095	RARRES2	-0.099	GDNF	-0.089	t-PA	-0.075
AZU1	-0.095	FGF-23	-0.098	RARRES2	-0.088	VEGFA	-0.072
SIRT2	-0.095	TLT-2	-0.097	CXCL16	-0.083	ST2	-0.069
RARRES2	-0.095	CTSZ	-0.097	TLT-2	-0.083	uPA	-0.069
TLT-2	-0.093	PI3	-0.096	RETN	-0.081	CXCL16	-0.069
AXIN1	-0.093	MMP-3	-0.094	TNF	-0.080	C5a	-0.069
PECAM-1	-0.092	CCL16	-0.093	IL2-RA	-0.080	MB	-0.066
IGFBP-2	-0.091	IL-6RA	-0.093	CXCL9	-0.080	IL18	-0.063
NT-proBNP	-0.091	Gal-4	-0.092	PD-L1	-0.080	TFF3	-0.062
MMP-3	-0.089	SHPS-1	-0.092	CD8A	-0.079	EN-RAGE	-0.061
IL-6RA	-0.089	Gal-3	-0.087	AXL	-0.077	FGF-21	-0.061
PLC	-0.087	HGF	-0.087	CSTB	-0.074	EPHB4	-0.061
PAI	-0.087	KLK6	-0.087	VEGFA	-0.073	TIMP-1	-0.060
IL8	-0.087	AXL	-0.087	TGF-alpha	-0.073	IL10	-0.060
PDGF subunit A	-0.085	CXCL9	-0.086	PSP-D	-0.073	MCP-4	-0.060
GDF-15	-0.085	CCL18 (PARC)	-0.085	CCL23	-0.072	TFPI	-0.059
TIMP4	-0.084	MB	-0.085	CPB1	-0.071	IL-17RA	-0.058
TR-AP	-0.083	TNF	-0.085	CDH5	-0.064	Gal-3	-0.056
CASP-3	-0.083	TNFRSF10C	-0.084	IL-6RA	-0.059	SHPS-1	-0.056
IL7	-0.081	MCP-3	-0.084	SLAMF1	-0.058	CSF-1	-0.055
BLM hydrolase	-0.081	DLK-1	-0.080	CCL3	-0.057	AP-N	-0.054
IL-18BP	-0.079	IL-17C	-0.079	CPA1	-0.056	IL-22 RA1	-0.053
LAP TGF-beta-1	-0.078	LIF-R	-0.079	IGFBP-1	-0.056	SCGB3A2	-0.051
FABP4	-0.078	FGF-5	-0.078	CHI3L1	-0.056	CCL19	-0.049
MMP-10	-0.078	JAM-A	-0.077	GRN	-0.055	IL-6RA	-0.049
Gal-3	-0.076	IL10	-0.076	MMP-2	-0.054	LTBR	-0.049
CCL20	-0.075	SCGB3A2	-0.074	SCGB3A2	-0.054	LDL receptor	-0.048
TR	-0.074	AZU1	-0.074	IL-12B	-0.053	IGFBP-7	-0.048
MCP-4	-0.073	IL6	-0.073	CDCP1	-0.052	TNFRSF14	-0.048
TNF	-0.072	FAS	-0.073	ICAM-2	-0.051	MMP-10	-0.048
CCL4	-0.072	TFPI	-0.073	CHIT1	-0.050	ADA	-0.046

ADA	-0.072	GRN	-0.070	IL33	-0.050	MCP-2	-0.045
MMP-1	-0.072	ICAM-2	-0.070	CNTN1	-0.049	CCL20	-0.045
IL-1RT2	-0.071	MMP-10	-0.070	IL-17C	-0.047	IL-15RA	-0.044
CD163	-0.071	GP6	-0.070	MMP-3	-0.047	FABP4	-0.042
IL-15RA	-0.069	OSM	-0.069	PON3	-0.047	IL-17A	-0.041
IL-1RT1	-0.069	Notch 3	-0.067	Ep-CAM	-0.045	CXCL10	-0.040
TFF3	-0.067	ALCAM	-0.067	PGLYRP1	-0.044	EGFR	-0.038
IL18	-0.066	IL-18R1	-0.066	ST2	-0.042	ICAM-2	-0.038
vWF	-0.066	IL18	-0.065	TNFRSF10C	-0.041	IL-2RB	-0.037
IL-17RA	-0.065	IL8	-0.065	TIMP-1	-0.041	IL2	-0.036
IGFBP-7	-0.065	SLAMF1	-0.065	ITGB2	-0.036	COL1A1	-0.036
ICAM-2	-0.065	4E-BP1	-0.062	IL-22 RA1	-0.036	IL2-RA	-0.035
MEPE	-0.065	TR-AP	-0.061	IL18	-0.034	PAI	-0.035
AXL	-0.065	CD8A	-0.061	IL-20	-0.031	OPN	-0.034
IL-17C	-0.064	TNFSF14	-0.061	Gal-3	-0.030	ITGB2	-0.033
IL-10RB	-0.064	CCL25	-0.060	MMP-10	-0.029	FGF-23	-0.032
TNFRSF9	-0.062	CXCL11	-0.059	IL13	-0.029	CCL15	-0.031
LIF	-0.061	CCL20	-0.058	CCL16	-0.029	IL-20RA	-0.029
CXCL9	-0.059	CD163	-0.054	COL1A1	-0.025	PLC	-0.027
FGF-23	-0.059	IL-24	-0.052	IL4	-0.024	TNFB	-0.027
CD93	-0.059	SELP	-0.051	IL-24	-0.023	CTSZ	-0.026
SCGB3A2	-0.056	AXIN1	-0.051	Beta-NGF	-0.021	MEPE	-0.024
IL-24	-0.055	CCL4	-0.050	CA15-3_MUC-1	-0.021	4E-BP1	-0.024
GRN	-0.054	STAMBP	-0.049	NT-3	-0.020	IGFBP-2	-0.023
SHPS-1	-0.054	MMP-9	-0.049	DNER	-0.019	GDNF	-0.023
CTSD	-0.053	OPG	-0.048	IL-10RA	-0.019	LAP TGF-beta-1	-0.022
CD5	-0.053	IGFBP-1	-0.048	IL10	-0.019	PDGF subunit A	-0.020
MCP-2	-0.051	CPB1	-0.048	TRANCE	-0.015	IL7	-0.020
CX3CL1	-0.051	IL-17RA	-0.048	ARTN	-0.013	Flt3L	-0.020
Gal-4	-0.050	MMP-2	-0.047	PCSK9	-0.012	NT-proBNP	-0.018
CTSZ	-0.050	CDCP1	-0.046	OPG	-0.012	LIF-R	-0.018
FAS	-0.049	CPA1	-0.046	NRTN	-0.011	CCL11	-0.017
PCSK9	-0.049	SIRT2	-0.045	MCP-1	-0.011	FGF-5	-0.017
PI3	-0.048	ADA	-0.045	IL5	-0.011	CPA1	-0.017
SLAMF1	-0.048	PCSK9	-0.044	IL-20RA	-0.009	CCL3	-0.016
OPG	-0.046	MCP-1	-0.041	CXCL10	-0.001	TNFSF13B	-0.016
MCP-1	-0.044	TR	-0.041	TFPI	0.000	SLAMF1	-0.013
TNFSF13B	-0.042	Beta-NGF	-0.041	CCL11	0.001	CD6	-0.012
AP-N	-0.041	PECAM-1	-0.041	FGF-21	0.001	CHIT1	-0.012
Beta-NGF	-0.040	IL-1RT2	-0.041	SELE	0.003	PI3	-0.008
MB	-0.040	IL-22 RA1	-0.037	TNFSF13B	0.003	IL-18BP	-0.007
t-PA	-0.039	vWF	-0.037	CCL28	0.005	CD93	-0.006
CXCL1	-0.039	PSP-D	-0.036	CD244	0.005	CD40	-0.004
CXCL10	-0.038	TNFSF13B	-0.036	TR-AP	0.006	PSP-D	-0.004
CXCL6	-0.036	CHIT1	-0.034	MCP-3	0.008	SELP	-0.002
CD244	-0.034	CASP-3	-0.033	CCL20	0.008	TLT-2	0.001
ST1A1	-0.034	CDH5	-0.033	CD163	0.008	CD5	0.001
LIF-R	-0.033	IL-20RA	-0.033	CD6	0.011	BLM hydrolase	0.001
CDCP1	-0.031	IL33	-0.030	IL-2RB	0.012	IL4	0.002
IL-20RA	-0.030	CXCL10	-0.028	IL-17RA	0.013	ALCAM	0.002
CASP-8	-0.030	IL-20	-0.027	CCL4	0.013	KLK6	0.002
IL-22 RA1	-0.029	CD244	-0.026	PRTN3	0.014	IFN-gamma	0.002
CCL28	-0.028	SCF	-0.025	C5a	0.014	IL-1RT1	0.002
IL-1 alpha	-0.027	LAP TGF-beta-1	-0.024	TRAIL	0.015	TR	0.003
FGF-21	-0.026	FGF-21	-0.023	EN-RAGE	0.015	TIMP4	0.003
FGF-5	-0.025	BLM hydrolase	-0.023	CCL18 (PARC)	0.016	CXCL9	0.004
TSLP	-0.024	CTSD	-0.023	CCL24	0.018	DLK-1	0.005
IGFBP-1	-0.024	ARTN	-0.023	LDL receptor	0.020	IL-10RB	0.006
CPA1	-0.023	NT-3	-0.022	Flt3L	0.021	MCP-3	0.008
CD8A	-0.022	IL-12B	-0.022	uPA	0.021	TWEAK	0.008
IL-17A	-0.021	CCL28	-0.020	JAM-A	0.024	ST1A1	0.008
SELE	-0.019	FGF-19	-0.019	IL8	0.025	CPB1	0.009
CHIT1	-0.018	MPO	-0.014	IL-1RT2	0.026	TRAIL	0.010
ARTN	-0.018	LIF	-0.013	TWEAK	0.027	IGFBP-1	0.010
IL-20	-0.018	SELE	-0.013	AZU1	0.027	CXCL1	0.013
ALCAM	-0.017	MCP-2	-0.011	TR	0.027	Notch 3	0.014
IL33	-0.015	IL-10RA	-0.010	IL-17A	0.029	CXCL6	0.015
IL-2RB	-0.014	IL4	-0.010	GP6	0.031	CDCP1	0.015
MMP-2	-0.013	Ep-CAM	-0.008	CXCL11	0.032	CCL4	0.023
CCL24	-0.012	IL7	-0.006	CCL19	0.032	SELE	0.024
CCL25	-0.011	IL-2RB	-0.006	vWF	0.033	PECAM-1	0.025
CPB1	-0.011	PDGF subunit A	-0.005	IL-18R1	0.033	IL-12B	0.025
IL-10RA	-0.009	CCL11	-0.004	IFN-gamma	0.037	CCL23	0.025
CST5	-0.009	CA15-3_MUC-1	-0.004	CASP-8	0.040	ARTN	0.025
NT-3	-0.008	COL1A1	-0.003	ADA	0.040	TNFRSF9	0.025
CCL19	-0.008	NRTN	-0.003	EGFR	0.040	CX3CL1	0.026
KLK6	-0.007	IL13	-0.003	IL2	0.042	NRTN	0.027
IL2	-0.005	IL5	-0.003	IL6	0.042	GP6	0.027
CXCL5	-0.005	MCP-4	-0.003	TSLP	0.045	CDH5	0.027
CCL11	-0.004	IL-17A	-0.003	CTSD	0.047	CD244	0.028
C5a	-0.002	MMP-1	-0.002	ST1A1	0.047	IL33	0.031
NRTN	-0.001	CCL24	-0.002	MCP-2	0.048	DNER	0.031

IL5	0.002	t-PA	-0.001	TNFB	0.048	JAM-A	0.032
IL13	0.003	ST1A1	-0.001	HGF	0.048	IL-20	0.033
PSP-D	0.004	CASP-8	0.004	4E-BP1	0.048	CASP-3	0.034
CDH5	0.005	CXCL6	0.004	AXIN1	0.049	TR-AP	0.039
IL4	0.005	AP-N	0.005	CXCL6	0.050	SIRT2	0.039
LDL receptor	0.005	CXCL1	0.007	CXCL5	0.050	CXCL11	0.041
CA15-3_MUC-1	0.009	TSLP	0.007	AP-N	0.051	CCL25	0.044
Notch 3	0.010	PAI	0.008	STAMBP	0.056	MCP-1	0.047
DLK-1	0.014	C5a	0.008	IL-1 alpha	0.057	IL5	0.049
TRAIL	0.014	IL-1 alpha	0.008	CXCL1	0.058	STAMBP	0.049
Ep-CAM	0.018	CCL19	0.012	t-PA	0.061	CST5	0.054
COL1A1	0.023	LDL receptor	0.014	LAP TGF-beta-1	0.061	Gal-4	0.054
TWEAK	0.026	PON3	0.016	SIRT2	0.062	AXIN1	0.058
uPA	0.028	TRAIL	0.018	CASP-3	0.063	CNTN1	0.059
EGFR	0.030	IL2	0.019	OSM	0.065	NT-3	0.062
IFN-gamma	0.032	CNTN1	0.026	PECAM-1	0.067	CA15-3_MUC-1	0.068
IL-12B	0.034	uPA	0.028	LIF	0.069	CXCL5	0.069
FGF-19	0.038	CXCL5	0.031	BLM hydrolase	0.071	IL13	0.075
CD6	0.043	ITGB2	0.032	SELP	0.074	TNF	0.093
TRANCE	0.052	TRANCE	0.033	MMP-9	0.075	IL-10RA	0.104
PON3	0.056	TWEAK	0.043	MMP-1	0.077	RARRES2	0.104
TNFB	0.062	IFN-gamma	0.043	MCP-4	0.080	Ep-CAM	0.114
ITGB2	0.065	DNER	0.047	TNFSF14	0.083	SCF	0.135
CNTN1	0.069	CD6	0.047	IL7	0.085	PON3	0.141
DNER	0.073	EGFR	0.052	PDGF subunit A	0.087	FGF-19	0.147
Flt3L	0.073	TNFB	0.068	MPO	0.104	CD8A	0.164
SCF	0.076	Flt3L	0.074	PAI	0.105	TRANCE	0.215

Loading scores represent the strength of each protein to separate the groups compared.

PLS-DA = partial least squares discriminant analysis; AAV = ANCA-associated vasculitis; PR3-AAV = Proteinase 3-ANCA positive vasculitis; MPO-AAV = Myeloperoxidase-ANCA positive vasculitis.

Supplementary table 5. Protein differential analysis: PR3-AAV vs. MPO-AAV.

AAV subtype with protein upregulation	Protein	Log2 FC	conf.low	conf.high	P_{adj}
**PR3-AAV**					
	MPO	0.59	0.096	1.1	0.0059
	PAI	0.96	0.086	1.8	0.018
	MMP-1	1.1	0.0061	2.2	0.047
	HGF	0.46	0.0002	0.92	0.05
	OSM	0.87	0.07	1.8	0.099
	MMP-9	0.84	-0.068	1.8	0.099
	IL-1RT2	0.33	-0.044	0.70	0.14
	TSLP	0.33	-0.069	0.73	0.21
	SELP	0.59	-0.14	1.3	0.24
	IL-18R1	0.34	-0.091	0.77	0.28
	IL7	0.56	-0.16	1.3	0.29
	LIF	0.37	-0.10	0.83	0.29
	IL8	0.51	-0.18	1.2	0.36
	MCP-4	0.52	-0.18	1.2	0.37
	TNFSF14	0.54	-0.20	1.3	0.39
	PDGF subunit A	0.62	-0.24	1.5	0.41
	CASP-8	0.59	-0.24	1.4	0.44
	t-PA	0.44	-0.20	1.1	0.50
	BLM hydrolase	0.30	-0.16	0.75	0.58
	AZU1	0.56	-0.35	1.5	0.65
	TFPI	0.21	-0.14	0.56	0.69
	LAP TGF-beta-1	0.30	-0.22	0.81	0.73
	CCL16	0.30	-0.22	0.81	0.75
	PRTN3	0.42	-0.33	1.2	0.76
	SIRT2	0.78	-0.65	2.2	0.80
	IL-1 alpha	0.16	-0.14	0.47	0.82
	CXCL6	0.43	-0.38	1.2	0.83
	STAMBP	0.64	-0.57	1.9	0.83
	PECAM-1	0.32	-0.31	0.96	0.86
	4E-BP1	0.51	-0.51	1.5	0.87
	AP-N	0.16	-0.17	0.49	0.88
	CXCL1	0.45	-0.47	1.4	0.89
	ST1A1	0.51	-0.58	1.6	0.92
	vWF	0.40	-0.48	1.3	0.93
	CASP-3	0.64	-0.77	2.1	0.93
	LDL receptor	0.24	-0.31	0.79	0.94
	ADA	0.21	-0.31	0.73	0.97
	IL6	0.45	-0.70	1.6	0.97
	EN-RAGE	0.40	-0.63	1.4	0.97
	MCP-2	0.26	-0.41	0.93	0.98
	EGFR	0.08	-0.13	0.29	0.98
	GP6	0.28	-0.46	1.0	0.98
	AXIN1	0.57	-0.93	2.1	0.98
	CTSD	0.15	-0.26	0.56	0.98
	OPG	0.11	-0.20	0.43	0.98
	MMP-3	0.33	-0.60	1.3	0.99
	CD163	0.17	-0.31	0.65	0.99
	IL-22 RA1	0.16	-0.28	0.6	0.99
	JAM-A	0.33	-0.60	1.3	0.99
	FGF-21	0.49	-0.92	1.9	0.99
	CXCL5	0.43	-0.85	1.7	0.99
	C5a	0.19	-0.43	0.82	1.0
	CCL11	0.14	-0.32	0.59	1.0
	CHI3L1	0.23	-0.55	1.0	1.0
	PCSK9	0.12	-0.30	0.54	1.0
	Gal-3	0.12	-0.32	0.57	1.0
	IL-2RB	0.16	-0.43	0.74	1.0
	TR	0.14	-0.41	0.70	1.0
	PGLYRP1	0.14	-0.43	0.70	1.0
	IL-6RA	0.08	-0.26	0.41	1.0
	IL-17C	0.12	-0.41	0.64	1.0
	CCL24	0.16	-0.60	0.93	1.0
	TWEAK	0.07	-0.26	0.41	1.0
	CCL28	0.08	-0.31	0.47	1.0
	TR-AP	0.11	-0.46	0.68	1.0
	CCL20	0.17	-0.74	1.1	1.0
	TNFRSF10C	0.08	-0.38	0.53	1.0
	ST2	0.12	-0.63	0.87	1.0
	CXCL11	0.14	-0.84	1.1	1.0
	Beta-NGF	0.04	-0.24	0.31	1.0
	TRAIL	0.050	-0.33	0.44	1.0
	CCL4	0.080	-0.53	0.68	1.0
	IL2	0.020	-0.16	0.20	1.0
	CPA1	0.090	-0.68	0.85	1.0
	CCL18_PARC	0.060	-0.49	0.61	1.0
	CXCL16	0.030	-0.27	0.34	1.0
	CD244	0.030	-0.35	0.41	1.0
	MCP-3	0.060	-0.65	0.76	1.0
	IL-17A	0.040	-0.62	0.70	1.0
	VEGFA	0.030	-0.40	0.45	1.0
	TNFB	0.020	-0.46	0.50	1.0
	IL10	0.020	-0.67	0.72	1.0
	FAS	0.010	-0.40	0.41	1.0
	IL-20RA	0.0047	-0.38	0.39	1.0
**MPO-AAV**					
	TNFRSF9	0.69	0.22	1.2	0.00014
	CST5	0.58	0.16	1.0	0.00051
	SCF	0.62	0.077	1.2	0.011
	CD5	0.37	0.026	0.71	0.023
	DLK-1	0.58	0.016	1.1	0.038

IL-10RB	0.26	0.0070	0.52	0.038
IL-18BP	0.35	0.0070	0.69	0.041
CX3CL1	0.38	-0.012	0.77	0.067
Notch 3	0.34	-0.019	0.69	0.081
TNF	0.53	-0.030	1.1	0.082
CD8A	0.60	-0.047	1.2	0.097
CD93	0.27	-0.027	0.56	0.11
NT-proBNP	0.96	-0.12	2.0	0.13
PLC	0.21	-0.046	0.47	0.23
LTBR	0.30	-0.081	0.68	0.28
KLK6	0.30	-0.087	0.69	0.30
CXCL9	0.61	-0.17	1.4	0.30
IL-12B	0.66	-0.20	1.5	0.31
MEPE	0.40	-0.14	0.94	0.38
IGFBP-2	0.39	-0.18	0.96	0.48
TNF-R2	0.29	-0.13	0.71	0.49
IGFBP-7	0.22	-0.10	0.55	0.49
SLAMF1	0.22	-0.10	0.54	0.49
PI3	0.38	-0.18	0.95	0.51
Gal-4	0.31	-0.15	0.77	0.51
FGF-23	0.50	-0.24	1.2	0.51
CTSZ	0.24	-0.12	0.60	0.55
IL-15RA	0.18	-0.098	0.47	0.57
TNFRSF14	0.29	-0.16	0.73	0.59
IL-1RT1	0.18	-0.10	0.46	0.60
LIF-R	0.18	-0.10	0.46	0.60
EPHB4	0.19	-0.11	0.49	0.61
TRANCE	0.38	-0.24	1.0	0.66
CNTN1	0.25	-0.16	0.65	0.66
TFF3	0.36	-0.23	0.95	0.67
IL2-RA	0.27	-0.18	0.73	0.69
PSP-D	0.43	-0.30	1.2	0.72
ALCAM	0.14	-0.11	0.39	0.76
FGF-5	0.12	-0.098	0.33	0.80
FGF-19	0.49	-0.43	1.4	0.82
TNF-R1	0.23	-0.20	0.66	0.83
TLT-2	0.22	-0.20	0.64	0.83
RARRES2	0.14	-0.15	0.43	0.88
PON3	0.31	-0.34	0.96	0.90
COL1A1	0.19	-0.22	0.60	0.92
CSF-1	0.087	-0.12	0.29	0.95
CDCP1	0.22	-0.31	0.75	0.95
OPN	0.23	-0.32	0.77	0.96
IL4	0.27	-0.39	0.92	0.96
CDH5	0.12	-0.18	0.42	0.97
NT-3	0.17	-0.30	0.63	0.98
U-PAR	0.16	-0.29	0.61	0.99
CHIT1	0.36	-0.65	1.4	0.99
Ep-CAM	0.33	-0.63	1.3	0.99
IGFBP-1	0.34	-0.71	1.4	0.99
IL33	0.074	-0.16	0.30	0.99
SHPS-1	0.11	-0.25	0.48	1.0
CD40	0.14	-0.33	0.61	1.0
CA15-3_MUC-1	0.19	-0.46	0.85	1.0
CCL25	0.13	-0.30	0.55	1.0
ICAM-2	0.10	-0.25	0.45	1.0
CCL23	0.11	-0.30	0.53	1.0
IL-20	0.10	-0.29	0.50	1.0
GDNF	0.093	-0.26	0.45	1.0
CCL15	0.094	-0.31	0.50	1.0
FABP4	0.16	-0.57	0.90	1.0
TIMP4	0.11	-0.41	0.63	1.0
MB	0.12	-0.44	0.69	1.0
ARTN	0.086	-0.32	0.49	1.0
GRN	0.067	-0.25	0.38	1.0
ITGB2	0.088	-0.33	0.51	1.0
RETN	0.12	-0.46	0.70	1.0
PD-L1	0.074	-0.31	0.46	1.0
IL5	0.12	-0.59	0.84	1.0
CSTB	0.12	-0.60	0.85	1.0
TNFSF13B	0.10	-0.47	0.67	1.0
IL-17RA	0.069	-0.35	0.49	1.0
CCL3	0.082	-0.45	0.61	1.0
CXCL10	0.14	-0.79	1.1	1.0
CCL19	0.13	-0.84	1.1	1.0
SELE	0.066	-0.47	0.60	1.0
IL18	0.070	-0.50	0.64	1.0
DNER	0.029	-0.21	0.27	1.0
NRTN	0.037	-0.27	0.34	1.0
MMP-2	0.044	-0.34	0.42	1.0
IL-10RA	0.060	-0.47	0.59	1.0
IL-24	0.060	-0.48	0.60	1.0
IL13	0.060	-0.53	0.65	1.0
MCP-1	0.055	-0.51	0.62	1.0
SPON1	0.020	-0.19	0.23	1.0
CD6	0.040	-0.47	0.55	1.0
Flt3L	0.035	-0.51	0.58	1.0
GDF-15	0.043	-0.69	0.77	1.0
CPB1	0.039	-0.66	0.74	1.0
TGF-alpha	0.020	-0.43	0.47	1.0
SCGB3A2	0.035	-0.77	0.84	1.0
uPA	0.012	-0.29	0.32	1.0
IFN-gamma	0.045	-1.2	1.3	1.0
MMP-10	0.017	-0.56	0.59	1.0
TIMP-1	0.0028	-0.35	0.36	1.0

AXL	0.0027	-0.30	0.31	1.0

PR3-AAV = Proteinase 3-ANCA positive vasculitis; MPO-AAV = Myeloperoxidase-ANCA positive vasculitis.;
Log2 FC = log2 fold change; P_{adj} = adjusted P value; Conf.low-conf.high = confidence interval (lower- higher).

Supplementary table 6. Sets of differentially expressed proteins based on the univariate and multivariate protein analyses in PR3-AAV and MPO-AAV.

Shared AAV-protein set (n = 21)	PR3-AAV protein set (n = 35)	MPO-AAV protein set (n = 33)	PR3-AAV-specific protein set (n = 17)	MPO-AAV-specific protein set (n = 23)
4E-BP1	4E-BP1	4E-BP1	AZU1	CCL3
AXIN1	AXIN1	AXIN1	CASP-3	CD40
BNP	AZU1	CCL18 (PARC)	CCL20	CD5
CCL18	CASP-3	CCL23	CHI3L1	CD8A
CCL23	CCL18 (PARC)	CCL3	GP6	CD93
CXCL11	CCL20	CD40	HGF	CSTB
EN-RAGE	CCL23	CSTB	IL10	CX3CL1
IL2-RA	CHI3L1	CXCL11	IL8	CXCL9
IL6	CXCL11	CXCL9	MMP-1	FGF-19
JAM-A	EN-RAGE	EN-RAGE	MMP-9	IGFBP-2
MCP-3	GP6	EPHB4	MPO	IL-10RB
OPN	IL10	IGFBP-2	OSM	IL-18BP
OSM	IL2-RA	IL2-RA	PAI	LTBR
PRTN3	IL6	IL6	PDGF subunit A	OPG
SIRT2	JAM-A	JAM-A	PGLYRP1	PLC
ST2	MCP-3	LTBR	SELP	RETN
STAMBP	MMP-1	MCP-3	TNFSF14	TNF
TIMP-1	MMP-9	NT-proBNP		TNF-R1
TNF-R1	NT-proBNP	OPN		TNF-R2
TNFRSF14	OPN	OSM		TNFRSF14
VEGF-A	OSM	PRTN3		TNFRSF9
	PAI	RETN		TRANCE
	PDGF subunit A	SIRT2		U-PAR
	PGLYRP1	ST2		
	PRTN3	STAMBP		
	SELP	TGF-alpha		
	SIRT2	TIMP-1		
	ST2	TNF		
	STAMBP	TNF-R1		
	TIMP-1	TNF-R2		
	TNF-R1	TNFRSF14		
	TNFRSF14	U-PAR		
	TNFSF14	VEGFA		
	U-PAR			
	VEGFA			

AAV = ANCA-associated vasculitis; PR3-AAV = Proteinase 3-ANCA positive vasculitis; MPO-AAV = Myeloperoxidase-ANCA positive vasculitis.

Supplementary table 7. Hub proteins across the AAV protein sets.

Shared AAV protein set		PR3-AAV protein set		PR3-AAV-specific protein set		MPO-AAV protein set		MPO-AAV-specific protein set	
**Protein**	**MCC value**	**Protein**	**MCC value**	**Protein**	**MCC value**	**Protein**	**MCC value**	**Protein**	**MCC value**
IL6	10.0	IL6	133.0	CXCL8	63.0	TNF	150.0	TNF	183.0
TNF-R1	5.0	MMP9	86.0	MMP9	60.0	IL6	126.0	TNF-R1	150.0
TIMP1	4.0	TIMP1	84.0	HGF	50.0	TNF-R1	80.0	CD40	150.0
IL2RA	4.0	IL10	78.0	MMP1	48.0	TNF-R2	72.0	TNFSF11	121.0
MCP-3	3.0	MMP1	48.0	IL10	40.0	CD40	54.0	TNF-R2	120.0

AAV = ANCA-associated vasculitis; PR3-AAV = Proteinase 3-ANCA positive vasculitis; MPO-AAV = Myeloperoxidase-ANCA positive vasculitis; MCC = maximal clique centrality.

Supplementary table 8. Biological processes and signaling pathways associated with the shared AAV protein set.

ID Ontology source	Enrichment term	P_{adj}	% Associated proteins	No. of proteins	Associated proteins found
KEGG:04061	Viral protein interaction with cytokine and cytokine receptor	5.0×10^{-13}	8	8	[CCL18. CCL23. MCP-3. CXCL11. IL2RA. IL6. TNFRSF14. TNF-R1]
WP:5095	Overview of proinflammatory and profibrotic mediators	3.6×10^{-12}	6	8	[CCL18. CCL23. MCP-3. CXCL11. IL6. OSM. OPN. VEGFA]
R-HSA:6789615	Expression of STAT3-upregulated extracellular proteins	3.7×10^{-8}	19	4	[IL6. OSM. TIMP1. VEGFA]
GO:0042531	Positive regulation of tyrosine phosphorylation of STAT protein	4.5×10^{-8}	7	5	[IL6. OSM. TIMP1. TNF-R1. VEGFA]
GO:0008009	Chemokine activity	1.2×10^{-6}	8	4	[CCL18. CCL23. MCP-3. CXCL11]
GO:1903140	Regulation of establishment of endothelial barrier	3.2×10^{-6}	17	3	[JAM-A. TNF-R1. VEGFA]
GO:0048245	Eosinophil chemotaxis	1.1×10^{-5}	10	3	[CCL18. CCL23. MCP-3]
WP:3617	Photodynamic therapy-induced NF-kB survival signaling	1.3×10^{-5}	9	3	[IL6. TNF-R1. VEGFA]
R-HSA:6783783	Interleukin-10 signaling	1.6×10^{-5}	6	3	[IL6. TIMP1. TNF-R1]
WP:2203	Thymic stromal lymphopoietin (TSLP) signaling pathway	1.6×10^{-5}	6	3	[EIF4EBP1. IL2RA. IL6]

ID ontology source = the reference database and specific identifier for each enrichment term; % associated proteins = percentage of proteins in the protein set associated with each enrichment term; nr. proteins = number of proteins in the protein set associated with each enrichment term; associated proteins found = specific proteins in the protein set linked to each enrichment term.

P_{adj}= adjusted P value; AAV = ANCA-associated vasculitis.

Supplementary table 9. Biological processes and signaling pathways associated with the PR3-AAV protein set. Top 15 terms according to adjusted P value.

ID Ontology Source	Enrichment term	P_{adj}	% Associated proteins	No. of proteins	Associated proteins found
KEGG:04061	Viral protein interaction with cytokine and cytokine receptor	3.1×10^{-16}	11	11	[CCL18. CCL20. CCL23. MCP-3. CXCL11. IL10. IL2RA. IL6. TNFRSF14. TNF-R1. TNFSF14]
WP:5095	Overview of proinflammatory and profibrotic mediators	5.5×10^{-15}	9	11	[CCL18. CCL20. CCL23. MCP-3. CXCL11. IL10. IL6. MMP1. MMP9. OSM. OPN]
GO:0071674	Mononuclear cell migration	1.5×10^{-11}	5	10	[CCL18. CCL20. CCL23. MCP-3. CXCL11. JAM-A. IL6. EN-RAGE. PAI. TNFRSF14]
R-HSA:6789615	Expression of STAT3-upregulated extracellular proteins	2.9×10^{-11}	29	6	[IL10. IL6. MMP1. MMP9. OSM. TIMP1]
GO:0002548	Monocyte chemotaxis	1.1×10^{-9}	10	7	[CCL18. CCL20. CCL23. MCP-3. IL6. EN-RAGE. PAI]
GO:0072676	Lymphocyte migration	1.8×10^{-8}	6	7	[CCL18. CCL20. CCL23. MCP-3. CXCL11. JAM-A. TNFRSF14]
GO:1990266	Neutrophil migration	2.9×10^{-8}	6	7	[CCL18. CCL20. CCL23. MCP-3. CXCL11. PRTN3. EN-RAGE]
R-HSA:6785047	IL10 positively regulates extracellular inflammatory mediators	1.3×10^{-7}	100	3	[IL10. TIMP1. TNF-R1]
KEGG:04657	IL-17 signaling pathway	3.4×10^{-7}	6	6	[CASP3. CCL20. MCP-3. IL6. MMP1. MMP9]
GO:0034112	Positive regulation of homotypic cell-cell adhesion	3.5×10^{-7}	25	4	[JAM-A. GP6. IL6. U-PAR]
R-HSA:6783783	Interleukin-10 signaling	4.2×10^{-7}	11	5	[CCL20. IL10. IL6. TIMP1. TNF-R1]
GO:0008009	chemokine activity	6.1×10^{-7}	10	5	[CCL18. CCL20. CCL23. MCP-3. CXCL11]
R-HSA:6785807	Interleukin-4 and Interleukin-13 signaling	6.6×10^{-7}	6	6	[IL10. IL6. MMP1. MMP9. OSM. TIMP1]
GO:0070555	Response to interleukin-1	7.1×10^{-7}	5	6	[CCL18. CCL20. CCL23. MCP-3. CHI3L1. IL6]
GO:0010543	Regulation of platelet activation	9.5×10^{-7}	9	5	[JAM-A. GP6. IL6. PDGF SUBUNIT A. SELP]

ID ontology source = the reference database and specific identifier for each enrichment term; % associated proteins = percentage of proteins in the protein set associated with each enrichment term; nr. proteins = number of proteins in the protein set associated with each enrichment term; associated proteins found = specific proteins in the protein set linked to each enrichment term.

P_{adj}= adjusted P value; PR3-AAV = Proteinase 3-ANCA positive vasculitis.

Supplementary table 10. Biological processes and signaling pathways associated with the MPO-AAV protein set. Top 15 terms according to adjusted P value.

ID Ontology Source	Enrichment term	P_{adj}	% Associated proteins	No. of proteins	Associated proteins found
KEGG:04061	Viral protein interaction with cytokine and cytokine receptor	1.9×10^{-20}	13	13	[CCL18. CCL23. CCL3. MCP-3. CXCL11. CXCL9. IL2RA. IL6. LTBR. TNF. TNFRSF14. TNF-R1. TNF-R2]
KEGG:04060	Cytokine-cytokine receptor interaction	2.4×10^{-19}	5	16	[CCL18. CCL23. CCL3. MCP-3. CD40. CXCL11. CXCL9. ST2. IL2RA. IL6. LTBR. OSM. TNF. TNFRSF14. TNF-R1. TNF-R2]
WP:5095	Overview of proinflammatory and profibrotic mediators	5.4×10^{-15}	9	11	[CCL18. CCL23. CCL3. MCP-3. CXCL11. CXCL9. IL6. OSM. OPN. TNF. VEGFA]
GO:0005125	Cytokine activity	7.6×10^{-13}	5	11	[CCL18. CCL23. CCL3. MCP-3. CXCL11. CXCL9. IL6. OSM. TIMP1. TNF. VEGFA]
GO:0071674	Mononuclear cell migration	1.5×10^{-11}	5	10	[CCL18. CCL23. CCL3. MCP-3. CXCL11. JAM-A. IL6. EN-RAGE. TNF. TNFRSF14]
GO:1990266	Neutrophil migration	6.6×10^{-10}	7	8	[CCL18. CCL23. CCL3. MCP-3. CXCL11. CXCL9. PRTN3. EN-RAGE]
GO:0050731	Positive regulation of peptidyl-tyrosine phosphorylation	3.7×10^{-9}	6	8	[CD40. IL6. OSM. TIMP1. TNF. TNFRSF14. TNF-R1. VEGFA]
R-HSA:6783783	Interleukin-10 signaling	6.3×10^{-9}	13	6	[CCL3. IL6. TIMP1. TNF. TNF-R1. TNF-R2]
GO:0007259	Receptor signaling pathway via JAK-STAT	7.9×10^{-9}	5	8	[CD40. IL6. OSM. STAMBP. TIMP1. TNF. TNF-R1. VEGFA]
GO:0008009	Chemokine activity	1.1×10^{-8}	12	6	[CCL18. CCL23. CCL3. MCP-3. CXCL11. CXCL9]
KEGG:04620	Toll-like receptor signaling pathway	1.9×10^{-8}	6	7	[CCL3. CD40. CXCL11. CXCL9. IL6. OPN. TNF]
GO:0072676	Lymphocyte migration	1.9×10^{-8}	6	7	[CCL18. CCL23. CCL3. MCP-3. CXCL11. JAM-A. TNFRSF14]
WP:3624	Lung fibrosis	3.5×10^{-8}	10	6	[CCL3. IL6. OPN. TGF-ALPHA. TIMP1. TNF]
WP:5088	Prostaglandin signaling	8.3×10^{-8}	15	5	[CCL3. CXCL9. IL6. TNF. VEGFA]
GO:0002548	Monocyte chemotaxis	8.4×10^{-8}	8	6	[CCL18. CCL23. CCL3. MCP-3. IL6. EN-RAGE]

ID ontology source = the reference database and specific identifier for each enrichment term; % associated proteins = percentage of proteins in the protein set associated with each enrichment term; nr. proteins = number of proteins in the protein set associated with each enrichment term; associated proteins found = specific proteins in the protein set linked to each enrichment term.
P_{adj}= adjusted P value; MPO-AAV = myeloperoxidase ANCA-positive vasculitis.

Supplementary table 11. Biological processes and signaling pathways associated with the PR3-AAV-specific protein set

ID Ontology Source	Enrichment term	P_{adj}	% Associated proteins	No. of proteins	Associated proteins found
R-HSA:6789615	Expression of STAT3-upregulated extracellular proteins	5.1×10^{-11}	24	5	[HGF. IL10. MMP1. MMP9. OSM]
R-HSA:6785807	Interleukin-4 and Interleukin-13 signaling	2.5×10^{-9}	6	6	[CXCL8. HGF. IL10. MMP1. MMP9. OSM]
KEGG:04657	IL-17 signaling pathway	1.1×10^{-7}	5	5	[CASP3. CCL20. CXCL8. MMP1. MMP9]
KEGG:05144	Malaria	6.3×10^{-7}	8	4	[CXCL8. HGF. IL10. SELP]
WP:3624	Lung fibrosis	1.4×10^{-6}	6	4	[CXCL8. HGF. MMP9. PDGF SUBUNIT A]
WP:2374	Oncostatin M signaling pathway	1.5×10^{-6}	6	4	[CASP3. MMP1. OSM. PAI]
R-HSA:6784160	IL10 negatively regulates extracellular inflammatory mediators	5.7×10^{-6}	12	3	[CCL20. CXCL8. IL10]
WP:3646	Hepatitis C and hepatocellular carcinoma	9.7×10^{-6}	6	3	[CASP3. CXCL8. MMP1]
WP:3617	Photodynamic therapy-induced NF-kB survival signaling	1.3×10^{-5}	8	3	[CXCL8. MMP1. MMP9]
KEGG:05219	Bladder cancer	1.6×10^{-5}	7	3	[CXCL8. MMP1. MMP9]
R-HSA:6783783	Interleukin-10 signaling	1.6×10^{-5}	6	3	[CCL20. CXCL8. IL10]

ID ontology source = the reference database and specific identifier for each enrichment term; % associated proteins =percentage of proteins in the protein set associated with each enrichment term; nr. proteins = number of proteins in the protein set associated with each enrichment term; associated proteins found = specific proteins in the protein set linked to each enrichment term.

P_{adj}= adjusted P value; PR3-AAV = proteinase 3 ANCA-positive vasculitis.

Supplementary table 12. Biological processes and signaling pathways associated with the MPO-AAV-specific protein set.

ID Ontology Source	Enrichment term	P_{adj}	% Associated proteins	No. of proteins	Associated proteins found
KEGG:04061	Viral protein interaction with cytokine and cytokine receptor	1.3×10^{-14}	9	9	[CCL3. CX3CL1. CXCL9. IL10RB. LTBR. TNF. TNFRSF14. TNF-R1. TNF-R2]
R-HSA:5668541	TNFR2 non-canonical NF-kB pathway	1.5×10^{-14}	8	9	[CD40. LTBR. TNF. TNFRSF11B. TNFRSF14. TNF-R1. TNF-R2. TNFRSF9. TNFSF11]
R-HSA:5669034	TNFs bind their physiological receptors	5.7×10^{-12}	21	6	[TNFRSF11B. TNFRSF14. TNF-R1. TNF-R2. TNFRSF9. TNFSF11]
R-HSA:5668534	cIAP1.2 ubiquitinates NIK in cIAP1.2:TRAF2::TRAF3:NIK	5.2×10^{-10}	21	5	[CD40. LTBR. TNF. TNF-R2. TNFSF11]
R-HSA:5668454	K63polyUb-cIAP1.2 ubiquitinates TRAF3	5.2×10^{-10}	21	5	[CD40. LTBR. TNF. TNF-R2. TNFSF11]
R-HSA:5668414	TRAF2 ubiquitinates cIAP1.2 in cIAP1.2:TRAF1:TRAF2:TRAF3:NIK	5.2×10^{-10}	21	5	[CD40. LTBR. TNF. TNF-R2. TNFSF11]
R-HSA:6783783	Interleukin-10 signaling	1.7×10^{-8}	11	5	[CCL3. IL10RB. TNF. TNF-R1. TNF-R2]
GO:0005035	Death receptor activity	2.5×10^{-8}	24	4	[TNF. TNFRSF14. TNF-R1. TNF-R2]
R-HSA:5669097	LTA trimer binds TNF-R1.1B.14	3.7×10^{-8}	75	3	[TNFRSF14. TNF-R1. TNF-R2]
R-HSA:5668481	Protesomal degradation of K48polyUb-TRAF3	5.9×10^{-8}	8	5	[CD40. LTBR. TNF. TNF-R2. TNFSF11]
R-HSA:5676594	TNF receptor superfamily (TNFSF) members mediating non-canonical NF-kB pathway	5.3×10^{-6}	17	3	[CD40. LTBR. TNFSF11]
GO:0150076	Neuroinflammatory response	7.8×10^{-6}	5	4	[CCL3. CX3CL1. TNF. TNF-R2]
WP:5088	Prostaglandin signaling	2.1×10^{-5}	9	3	[CCL3. CXCL9. TNF]
WP:453	Inflammatory response pathway	2.1×10^{-5}	9	3	[CD40. TNF-R1. TNF-R2]
R-HSA:380108	Chemokine receptors bind chemokines	4.1×10^{-5}	5	3	[CCL3. CX3CL1. CXCL9]
GO:0001774	Microglial cell activation	4.4×10^{-5}	6	3	[CCL3. CX3CL1. TNF]

ID ontology source = the reference database and specific identifier for each enrichment term; % associated proteins = percentage of proteins in the protein set associated with each enrichment term; nr. proteins = number of proteins in the protein set associated with each enrichment term; associated proteins found = specific proteins in the protein set linked to each enrichment term.
P_{adj}= adjusted P value; MPO-AAV = myeloperoxidase ANCA-positive vasculitis.

Supplementary table 13. Proteins with significant differential expression, RA and SLE vs population controls.

Disease control group	Protein	Log2 FC	conf.low	conf.high	P_{adj}
**RA**					
	IL6	3.7	2.9	4.6	<1x10^-12
	EN-RAGE	3.2	2.4	4.0	<1x10^-12
	SIRT2	3.0	1.9	4.0	<1x10^-12
	STAMBP	2.5	1.6	3.4	<1x10^-12
	MMP-3	2.3	1.6	3.0	<1x10^-12
	MCP-3	2.1	1.5	2.6	<1x10^-12
	CXCL11	2.0	1.3	2.8	<1x10^-12
	MMP-1	2.0	1.2	2.8	<1x10^-12
	JAM-A	1.9	1.2	2.6	<1x10^-12
	4E-BP1	1.9	1.1	2.7	<1x10^-12
	SELP	1.7	1.1	2.2	<1x10^-12
	CXCL10	1.7	0.97	2.4	<1x10^-12
	GP6	1.6	1.1	2.2	<1x10^-12
	PDGF subunit A	1.5	0.89	2.2	<1x10^-12
	TNFSF14	1.5	0.89	2.0	<1x10^-12
	CHI3L1	1.4	0.80	2.0	<1x10^-12
	PECAM-1	1.2	0.74	1.7	<1x10^-12
	VEGFA	1.0	0.70	1.3	<1x10^-12
	CCL18 (PARC)	1.0	0.59	1.4	<1x10^-12
	AXIN1	2.6	1.4	3.7	5.8x10^-11
	CASP-3	2.4	1.3	3.4	1.6x10^-10
	IL7	1.2	0.65	1.7	3.7x10^-10
	CXCL9	1.2	0.61	1.8	9.0x10^-09
	IL8	1.1	0.56	1.6	4.3x10^-09
	PAI	1.3	0.67	2.0	1.1x10^-08
	CCL19	1.4	0.62	2.1	2.9x10^-07
	CCL20	1.3	0.61	2.0	1.3x10^-07
	CXCL1	1.2	0.54	1.9	9.1x10^-07
	IL10	1.0	0.47	1.5	1.1x10^-07
	CXCL6	1.0	0.40	1.6	8.4x10^-06
	ST1A1	1.1	0.31	2.0	0.00057
	OSM	1.0	0.31	1.7	0.00026
	CXCL5	1.3	0.29	2.2	0.0015
**SLE**					
	CXCL10	2.4	1.4	3.4	<1.0x10^-12
	TNF	2.3	1.7	2.9	<1.0x10^-12
	CCL3	2.00	1.5	2.6	<1.0x10^-12
	MCP-3	2.00	1.3	2.8	<1.0x10^-12
	PRTN3	2.00	1.2	2.8	<1.0x10^-12
	TNF-R2	2.00	1.6	2.5	<1.0x10^-12
	IL10	2.00	1.3	2.7	<1.0x10^-12
	IGFBP-2	1.7	1.1	2.3	<1.0x10^-12
	CCL4	1.6	0.98	2.3	<1.0x10^-12
	IL2-RA	1.5	1.1	2.0	<1.0x10^-12
	CD163	1.4	0.92	2.0	<1.0x10^-12
	TNF-R1	1.4	0.91	1.8	<1.0x10^-12
	MPO	1.3	0.80	1.8	<1.0x10^-12
	CX3CL1	1.3	0.84	1.7	<1.0x10^-12
	TNFRSF9	1.2	0.69	1.7	<1.0x10^-12
	IL-18BP	1.1	0.76	1.5	<1.0x10^-12
	PD-L1	1.1	0.66	1.5	<1.0x10^-12
	AXL	1.00	0.69	1.4	<1.0x10^-12
	TIMP-1	1.00	0.64	1.4	<1.0x10^-12
	GRN	1.00	0.67	1.3	<1.0x10^-12
	CXCL9	1.8	0.97	2.6	7.5x10^-10
	MCP-1	1.4	0.74	2.0	1.7x10^-10
	U-PAR	1.00	0.53	1.5	2.1x10^-09
	NT-proBNP	2.3	1.1	3.4	4.0x10^-08
	CXCL11	2.1	1.1	3.2	1.1x10^-08
	OPN	1.2	0.60	1.8	1.1x10^-08
	CDCP1	1.1	0.55	1.7	2.9x10^-08
	ST2	1.5	0.69	2.3	2.2x10^-07
	RETN	1.1	0.50	1.7	6.2x10^-07
	IFN-gamma	2.1	0.84	3.4	8.3x10^-06
	IL6	2.1	0.83	3.3	6.0x10^-06
	EN-RAGE	1.8	0.74	2.9	6.7x10^-6
	IL18	1.1	0.45	1.7	1.7x10^-06
	TR	1.00	0.42	1.6	3.5x10^-6
	CCL19	1.6	0.54	2.6	7.4x10^-5
	CHI3L1	1.3	0.50	2.2	2.0x10^-5
	MCP-2	1.2	0.46	1.9	1.2x10^-5
	IL8	1.2	0.43	1.9	2.8x10^-5
	TRANCE	1.00	0.33	1.7	9.3x10^-5
	IL-12B	1.2	0.24	2.1	0.0028
	IGFBP-1	1.2	0.11	2.3	0.019

RA = rheumatoid arthritis; SLE = Systemic lupus erythematosus; Log2 FC = log2 fold change; Conf.low-conf.high = confidence interval (lower- higher); P_{adj} = adjusted P value.

Supplementary table 14. Differentially expressed proteins, PR3-AAV vs. RA and SLE.

Group with protein upregulation	Protein	Log2 FC	conf.low	conf.high	P_{adj}
**PR3-AAV vs. RA**					
	OSM	1.0	0.13	1.9	0.0097
**RA vs. PR3-AAV**					
	MMP-3	1.4	0.54	2.2	1.1×10^{-5}
	IL6	1.4	0.29	2.4	0.0019
	EN-RAGE	1.2	0.27	2.1	0.0017
	FGF-19	1.1	0.28	1.9	0.00091
	CXCL10	1.0	0.17	1.8	0.0052
	CCL19	1.0	0.13	1.9	0.010
**PR3-AAV vs. SLE**					
	AXIN1	2.0	0.21	3.7	0.015
	SIRT2	1.9	0.24	3.61	0.010
	STAMBP	1.7	0.3	3.2	0.0048
	OSM	1.6	0.48	2.7	0.00025
	MMP-1	1.4	0.16	2.7	0.014
	JAM-A	1.3	0.20	2.4	0.0066
	MMP-9	1.3	0.19	2.3	0.0076
	SELP	1.2	0.30	2.0	0.00075
	PAI	1.2	0.13	2.2	0.013
	PDGF subunit A	1.0	0.010	2.0	0.045
**SLE vs. PR3-AAV**					
	IFN-gamma	2.4	0.98	3.8	4.5×10^{-6}
	CXCL10	1.8	0.69	2.9	1.2×10^{-5}
	TNF	1.7	1.03	2.3	1.0×10^{-13}
	IL-12B	1.5	0.51	2.5	8.3×10^{-5}
	TRANCE	1.4	0.65	2.1	1.4×10^{-7}
	TNF-R2	1.3	0.76	1.7	1.0×10^{-12}
	CCL19	1.2	0.10	2.4	0.020
	CX3CL1	1.2	0.74	1.7	1.0×10^{-12}
	CXCL9	1.1	0.19	2.0	0.0051
	CCL3	1.1	0.48	1.7	9.9×10^{-7}
	IL10	1.0	0.18	1.8	0.0041

PR3-AAV = proteinase 3 ANCA-positive vasculitis; RA = rheumatoid arthritis; SLE = Systemic lupus erythematosus; Log2 FC = log2 fold change; Conf.low-conf.high = confidence interval (lower- higher); P_{adj} = adjusted P value.

Supplementary table 15. Differentially expressed proteins, MPO-AAV vs. RA and SLE.

Group with protein upregulation	Protein	Log2 FC	conf.low	conf.high	P_{adj}
**MPO-AAV vs RA**					
	NT-proBNP	1.5	0.30	2.7	0.0030
**RA vs MPO-AAV**					
	MMP-1	1.9	0.70	3.1	2.9×10^{-5}
	IL6	1.8	0.51	3.1	0,.00040
	MMP-3	1.7	0.69	2.8	7.7×10^{-6}
	EN-RAGE	1.6	0.47	2.8	0.00036
	CXCL5	1.5	0.047	2.9	0.036
	CXCL1	1.2	0.15	2.2	0.010
	PAI	1.1	0.17	2.1	0.0078
	PDGF subunit A	1.1	0.14	2.1	0.011
	CASP-8	1.0	0.087	1.9	0.019
	CXCL6	1.0	0.11	1.9	0.015
**SLE vs MPO-AAV**					
	IFN-gamma	2.3	0.75	4.0	0.00016
	CXCL10	1.6	0.4	2.9	0.0011
	TNF	1.2	0.42	1.9	3.4×10^{-5}
	MPO	1.1	0.44	1.7	5.9×10^{-6}
	CD163	1.1	0.43	1.7	5.9×10^{-6}
	IL10	1.0	0.1	2.0	0.016
	CCL3	1.0	0.32	1.7	0.00018
	TRANCE	1.0	0.17	1.8	0.0052

MPO-AAV = myeloperoxidase ANCA-positive vasculitis; RA = rheumatoid arthritis; SLE = Systemic lupus erythematosus; Log2 FC = log2 fold change; P_{adj} = adjusted P value; Conf.low-conf.high = confidence interval (lower- higher).
